# Supplementary material for: Genome-Wide Association Study Identifies Genetic Polymorphisms Associated with Estimated Minimum Effective Concentration of Fentanyl in Patients Undergoing Laparoscopic-Assisted Colectomy
Source: Int J Mol Sci. 2023 May 8;24(9):8421. doi: 10.3390/ijms24098421 (PMC10179231; doi:10.3390/ijms24098421)
Supplement: Supplementary file 1 [file ijms-24-08421-s001.zip › ijms-2361651-supplementary.pdf]

International Journal of
*Molecular Sciences*

MDPI

*Supplementary Materials*

# Genome-Wide Association Study Identifies Genetic Polymorphisms Associated with Estimated Minimal Effective Analgesic Concentration of Fentanyl in Patients Undergoing Laparoscopic-Assisted Colectomy

**Daisuke Nishizawa, Tsutomu Mieda, Miki Tsujita, Hideyuki Nakagawa, Shigeki Yamaguchi, Shinya Kasai, Junko Hasegawa, Kyoko Nakayama, Yuko Ebata, Akira Kitamura, Hirotomo Shimizu, Tadayuki Takashima, Masakazu Hayashida and Kazutaka Ikeda**

**Table S1.** Demographic and clinical data of subjects who underwent laparoscopic-assisted colectomy.

| | *n* | Minimum | Maximum | Mean | SD | Median |
|---|---|---|---|---|---|---|
| **Sex** | | | | | | |
| male | 218 | | | | | |
| female | 133 | | | | | |
| **Age** | 351 | 22 | 85 | 63.72 | 10.44 | 65.00 |
| **Body height (cm)** | 351 | 137 | 184 | 160.87 | 9.23 | 161.00 |
| **Body weight (kg)** | 351 | 35 | 99 | 60.17 | 11.02 | 60.00 |
| **Types of laparoscopic assisted surgery** | | | | | | |
| Colectomy | 189 | | | | | |
| Proctectomy | 160 | | | | | |
| Coloproctectomy | 2 | | | | | |
| **Duration of surgery (min)** | 351 | 74.00 | 487.00 | 210.60 | 74.89 | 206.00 |
| **Duration of anesthesia (min)** | 351 | 128.00 | 554.00 | 275.15 | 77.65 | 273.00 |
| **Average remifentanil infusion rate** | 351 | 0.04 | 0.61 | 0.22 | 0.07 | 0.22 |
| **Average BIS**[†] | 347 | 29.6 | 69.10 | 46.89 | 7.85 | 45.50 |
| **Dose of fentanyl at the end of surgery (μg)** | 351 | 40 | 600.00 | 168.28 | 150.00 | 3.46 |
| **6-h PCA fentanyl consumption (μg/kg)** | 350 | 0 | 20.41 | 4.15 | 3.13 | 3.46 |
| **12-h PCA fentanyl consumption (μg/kg)** | 350 | 0 | 20.89 | 6.08 | 4.30 | 5.26 |
| **24-h PCA fentanyl consumption (μg/kg)** | 350 | 0 | 23.81 | 9.56 | 5.15 | 9.36 |
| **6-h total postoperative fentanyl requirement (μg/kg)** | 350 | 1.59 | 24.49 | 6.96 | 3.54 | 6.48 |
| **12-h total postoperative fentanyl requirement (μg/kg)** | 350 | 1.64 | 24.56 | 8.90 | 4.67 | 8.11 |
| **24-h total postoperative fentanyl requirement (μg/kg)** | 350 | 1.79 | 28.07 | 12.38 | 5.50 | 12.06 |
| **0-6 h plasma MEC (ng/ml)** | 350 | 0.03 | 3.64 | 0.92 | 0.57 | 0.82 |
| **0-12 h plasma MEC (ng/ml)** | 350 | 0.03 | 3.19 | 0.86 | 0.52 | 0.78 |
| **0-24 h plasma MEC (ng/ml)** | 350 | 0.03 | 2.44 | 0.69 | 0.39 | 0.64 |
| **0-6 h effect Site MEC (ng/ml)** | 350 | 0.03 | 2.97 | 0.75 | 0.43 | 0.69 |
| **0-12 h effect Site MEC (ng/ml)** | 350 | 0.03 | 2.66 | 0.72 | 0.41 | 0.66 |
| **0-24 h effect Site MEC (ng/ml)** | 350 | 0.03 | 3.06 | 0.79 | 0.48 | 0.72 |
| **6-h numbers of doses locked out** | 350 | 0 | 222.00 | 10.38 | 23.18 | 2.00 |
| **12-h numbers of doses locked out** | 349 | 0 | 249.00 | 12.71 | 27.60 | 3.00 |
| **24-h numbers of doses locked out** | 348 | 0 | 249.00 | 15.64 | 29.35 | 5.00 |
| **Postoperative pain score (2 h)** | 345 | 0 | 10.00 | 3.90 | 2.15 | 4.00 |
| **Postoperative pain score (4 h)** | 328 | 0 | 8.00 | 3.00 | 1.96 | 3.00 |
| **Postoperative pain score (6 h)** | 311 | 0 | 8.00 | 2.62 | 1.89 | 3.00 |
| **Postoperative pain score (12 h)** | 298 | 0 | 8.00 | 2.18 | 1.87 | 2.00 |
| **Postoperative pain score (24 h)** | 290 | 0 | 12.00 | 2.09 | 1.76 | 2.00 |
| **Maximum postoperative pain scores (2 h - 12 h)** | 350 | 0 | 10.00 | 4.26 | 2.01 | 4.00 |
| **Maximum postoperative pain scores (2 h - 24 h)** | 350 | 0 | 10.00 | 4.44 | 1.99 | 4.50 |
| **Average respiratory rate (2 h, 4 h, 6 h)** | 341 | 9.67 | 29.67 | 16.37 | 3.16 | 16.00 |
| **Sedation score (2 h - 24 h)** | 311 | | | | | |
| 0 | 276 | | | | | |
| > 0 | 35 | | | | | |
| **PONV**[§] **score (2 h - 24 h)** | 319 | | | | | |
| 0 | 235 | | | | | |
| > 0 | 84 | | | | | |
| **24-h postoperative rescue analgesics** | 351 | | | | | |
| non-use | 192 | | | | | |
| use | 159 | | | | | |

[†]Bispectral index (BIS); [‡]Minimum effective concentration (MEC); [§]Postoperative nausea and vomiting (PONV).

**Table S2.** Top candidate SNPs selected from three-stage GWAS for the 0-12 h plasma MEC.

| Model | Rank | SNP | CHR | Position | 1st stage | | 2nd stage | | Final stage | | | Combined | | Related gene |
|---|---|---|---|---|---|---|---|---|---|---|---|---|---|---|
| | | | | | *β* | *p* | *β* | *p* | *β* | *p* | *q* | *β* | *p* | |
| Additive | 1 | rs966775 | 5 | 174763322 | 0.0959 | 0.0032 | 0.0793 | 0.0293 | 0.0968 | 0.0001 | 0.0568 | 0.09081 | 1.21E-07 | (*DRD1*) |
| Additive | 2 | rs9354118 | 6 | 95147902 | 0.0508 | 0.0304 | 0.0619 | 0.0211 | 0.0627 | 0.0163 | 0.6848 | 0.05946 | 3.63E-05 | - |
| Additive | 3 | rs43211 | 19 | 54652203 | -0.0526 | 0.0246 | -0.0851 | 0.0047 | -0.0559 | 0.0337 | 0.7043 | -0.06048 | 4.21E-05 | *CNOT3* |
| Additive | 4 | rs9342409 | 6 | 95098682 | 0.0496 | 0.0328 | 0.0629 | 0.0254 | 0.0598 | 0.0229 | - | 0.05884 | 5.56E-05 | - |
| Additive | 5 | rs4806716 | 19 | 54639868 | -0.0545 | 0.0239 | -0.0657 | 0.0257 | -0.0596 | 0.0227 | 0.6848 | -0.05883 | 8.06E-05 | - |
| Additive | 6 | rs9363197 | 6 | 95104559 | 0.0508 | 0.0304 | 0.055 | 0.0451 | 0.0598 | 0.0229 | - | 0.057 | 8.48E-05 | - |
| Additive | 7 | rs4764074 | 12 | 14428118 | -0.0738 | 0.0185 | -0.063 | 0.0342 | -0.0588 | 0.0368 | 0.7043 | -0.06532 | 9.8E-05 | - |
| Additive | 8 | rs12035559 | 1 | 34499921 | -0.0842 | 0.0327 | -0.072 | 0.0158 | -0.0564 | 0.0444 | 0.7043 | -0.06822 | 0.000103 | *CSMD2* |
| Additive | 9 | rs10486791 | 7 | 16284326 | 0.0656 | 0.0395 | 0.0783 | 0.0394 | 0.0932 | 0.0186 | 0.6848 | 0.07521 | 0.000191 | *ISPD,LOC100506025* |
| Additive | 10 | rs2160974 | 12 | 108883621 | 0.0541 | 0.0352 | 0.0642 | 0.0289 | 0.0648 | 0.0166 | 0.6848 | 0.05566 | 0.000342 | - |
| Additive | 11 | rs936229 | 15 | 75132319 | 0.0748 | 0.0194 | 0.0657 | 0.0294 | 0.0635 | 0.0399 | 0.7043 | 0.06289 | 0.000345 | *ULK3* |
| Additive | 12 | rs9643154 | 8 | 122197394 | 0.0601 | 0.0446 | 0.0695 | 0.0467 | 0.075 | 0.0392 | 0.7043 | 0.06558 | 0.00054 | - |
| Additive | 13 | rs652930 | 1 | 201628577 | 0.0877 | 0.0323 | 0.1019 | 0.0389 | 0.1756 | 0.0129 | 0.6778 | 0.09816 | 0.000559 | *NAV1* |
| Additive | 14 | rs13329602 | 15 | 47472264 | 0.079 | 0.0427 | 0.1683 | 0.0254 | 0.1291 | 0.0296 | - | 0.09806 | 0.000911 | - |
| Dominant | 1 | rs9533839 | 13 | 44961158 | 0.0841 | 0.0213 | 0.1232 | 0.0013 | 0.08 | 0.0439 | - | 0.09564 | 9.25E-06 | *SERP2* |
| Dominant | 2 | rs6502266 | 17 | 13395720 | 0.1133 | 0.0024 | 0.0963 | 0.0155 | 0.0803 | 0.0289 | 0.7906 | 0.09311 | 1.17E-05 | - |
| Dominant | 3 | rs9889837 | 17 | 13392473 | 0.1133 | 0.0024 | 0.0963 | 0.0155 | 0.0753 | 0.0406 | - | 0.0919 | 1.52E-05 | - |
| Dominant | 4 | rs17081058 | 13 | 25267734 | -0.0998 | 0.0104 | -0.0966 | 0.0215 | -0.09 | 0.0283 | 0.7906 | -0.09937 | 1.57E-05 | *ATP12A* |
| Dominant | 5 | rs9968875 | 6 | 151313367 | 0.1933 | 0.0227 | 0.2025 | 0.002 | 0.192 | 0.0335 | 0.7906 | 0.1866 | 1.93E-05 | *MTHFD1L* |
| Dominant | 6 | rs746427 | 20 | 48939076 | -0.0833 | 0.0249 | -0.0925 | 0.0348 | -0.0984 | 0.0108 | - | -0.09138 | 3.3E-05 | - |
| Dominant | 7 | rs12868519 | 13 | 44972629 | 0.0748 | 0.0419 | 0.1138 | 0.0034 | 0.08 | 0.0439 | - | 0.0887 | 4.34E-05 | - |
| Dominant | 8 | rs17738087 | 15 | 26905021 | -0.0988 | 0.0201 | -0.1016 | 0.0233 | -0.0979 | 0.0093 | 0.7906 | -0.09569 | 4.43E-05 | *GABRB3* |
| Dominant | 9 | rs10836454 | 11 | 4696875 | -0.1065 | 0.0302 | -0.0909 | 0.0462 | -0.1265 | 0.0038 | 0.7906 | -0.1058 | 4.47E-05 | - |
| Dominant | 10 | rs4131101 | 5 | 119195837 | 0.1005 | 0.0145 | 0.1135 | 0.0128 | 0.0815 | 0.0479 | 0.7906 | 0.09806 | 5.09E-05 | - |
| Dominant | 11 | rs1431210 | 6 | 103229346 | 0.1076 | 0.0173 | 0.1004 | 0.0161 | 0.0865 | 0.0294 | - | 0.09545 | 5.86E-05 | - |
| Dominant | 12 | rs6020445 | 20 | 48939863 | -0.0833 | 0.0249 | -0.0977 | 0.0269 | -0.0859 | 0.0279 | 0.7906 | -0.08859 | 6.07E-05 | - |
| Dominant | 13 | rs751687 | 8 | 15608896 | 0.1023 | 0.0053 | 0.0891 | 0.0235 | 0.0766 | 0.0365 | 0.7906 | 0.08549 | 6.3E-05 | *TUSC3* |
| Dominant | 14 | rs4580854 | 6 | 15025298 | -0.0797 | 0.0335 | -0.093 | 0.0175 | -0.0848 | 0.0217 | 0.7906 | -0.08524 | 6.77E-05 | - |
| Dominant | 15 | rs1931550 | 6 | 12362219 | -0.0924 | 0.0479 | -0.0926 | 0.035 | -0.1143 | 0.0083 | 0.7906 | -0.1005 | 6.88E-05 | - |
| Dominant | 16 | rs1160226 | 13 | 25271434 | -0.0803 | 0.0361 | -0.097 | 0.0166 | -0.0786 | 0.0367 | 0.7906 | -0.08682 | 7.15E-05 | *ATP12A* |
| Dominant | 17 | rs6481157 | 10 | 57099471 | -0.1197 | 0.0024 | -0.084 | 0.0334 | -0.0752 | 0.0464 | 0.7906 | -0.08606 | 7.57E-05 | - |
| Dominant | 18 | rs13195313 | 6 | 103175290 | 0.1021 | 0.0204 | 0.0943 | 0.0246 | 0.0899 | 0.0257 | - | 0.09351 | 8.34E-05 | - |
| Dominant | 19 | exm2270377 | 6 | 103225137 | 0.1076 | 0.0173 | 0.0943 | 0.0246 | 0.0865 | 0.0294 | - | 0.09331 | 8.62E-05 | - |
| Dominant | 20 | rs13278423 | 8 | 87720419 | -0.1088 | 0.009 | -0.0891 | 0.0428 | -0.0933 | 0.0205 | 0.7906 | -0.09299 | 0.000104 | *CNGB3* |
| Dominant | 21 | rs3935993 | 5 | 119196820 | 0.086 | 0.0326 | 0.1135 | 0.0128 | 0.0815 | 0.0479 | - | 0.09191 | 0.000133 | - |
| Dominant | 22 | rs1027804 | 8 | 18919857 | -0.0864 | 0.0262 | -0.0829 | 0.0447 | -0.1042 | 0.0068 | 0.7906 | -0.08533 | 0.000148 | - |
| Dominant | 23 | rs10956972 | 8 | 87768331 | 0.0947 | 0.0204 | 0.0913 | 0.0267 | 0.1005 | 0.0136 | - | 0.08681 | 0.000189 | - |
| Dominant | 23 | rs1982563 | 8 | 87776019 | 0.0947 | 0.0204 | 0.0913 | 0.0267 | 0.1005 | 0.0136 | 0.7906 | 0.08681 | 0.000189 | - |
| Dominant | 25 | rs7592517 | 2 | 76777279 | 0.116 | 0.0047 | 0.0945 | 0.022 | 0.0809 | 0.0348 | - | 0.08232 | 0.0002 | - |
| Dominant | 25 | rs2139502 | 2 | 76786845 | 0.116 | 0.0047 | 0.0945 | 0.022 | 0.0809 | 0.0348 | 0.7906 | 0.08232 | 0.0002 | - |
| Dominant | 27 | rs4963573 | 12 | 24662116 | -0.0799 | 0.0314 | -0.0832 | 0.0337 | -0.0739 | 0.0452 | 0.7906 | -0.07812 | 0.000243 | *SOX5* |
| Dominant | 28 | rs12580224 | 12 | 71086426 | 0.0953 | 0.0138 | 0.0855 | 0.0393 | 0.0815 | 0.0387 | 0.7906 | 0.07883 | 0.000409 | *PTPRR* |
| Recessive | 1 | rs966775 | 5 | 174763322 | 0.1661 | 0.0082 | 0.165 | 0.0187 | 0.1631 | 0.0006 | 0.1216 | 0.1626 | 6.68E-07 | (*DRD1*) |
| Recessive | 2 | rs1728200 | 12 | 17615669 | 0.1758 | 0.0098 | 0.2811 | 0.0014 | 0.1855 | 0.0442 | 0.7049 | 0.2204 | 1.38E-06 | - |
| Recessive | 3 | rs43211 | 19 | 54652203 | -0.0859 | 0.0374 | -0.1449 | 0.0098 | -0.13 | 0.0052 | 0.4457 | -0.1114 | 2.45E-05 | *CNOT3* |
| Recessive | 4 | rs4764074 | 12 | 14428118 | -0.1282 | 0.0325 | -0.1187 | 0.0335 | -0.1328 | 0.0114 | 0.5034 | -0.125 | 7.78E-05 | - |
| Recessive | 5 | rs2146423 | 9 | 4657040 | 0.1221 | 0.0298 | 0.1254 | 0.0388 | 0.1648 | 0.0086 | 0.4457 | 0.1265 | 0.000163 | *C9orf68* |
| Recessive | 6 | rs10486791 | 7 | 16284326 | 0.1288 | 0.034 | 0.1495 | 0.0425 | 0.1735 | 0.0242 | 0.6709 | 0.1431 | 0.00024 | *ISPD,LOC100506025* |
| Recessive | 7 | rs2759632 | 10 | 10218843 | -0.1806 | 0.0282 | -0.2059 | 0.0316 | -0.1576 | 0.0436 | 0.7049 | -0.1759 | 0.000263 | - |
| Recessive | 8 | rs2368473 | 17 | 32534215 | 0.2993 | 0.0417 | 0.1602 | 0.0367 | 0.2285 | 0.0474 | 0.7049 | 0.175 | 0.001992 | - |

CHR, chromosome number; Position, chromosomal position (bp); *q*, *q* value for FDR correction of multiple comparison; Related gene, nearest gene from the SNP site.

**Table S3.** Top candidate SNPs selected from three-stage GWAS for the 0-6 h effect site MEC.

| Model | Rank | SNP | CHR | Position | 1st stage | | 2nd stage | | Final stage | | | Combined | | Related gene |
|---|---|---|---|---|---|---|---|---|---|---|---|---|---|---|
| | | | | | *β* | *p* | *β* | *p* | *β* | *p* | *q* | *β* | *p* | |
| Additive | 1 | rs966775 | 5 | 174763322 | 0.1123 | 0.0061 | 0.0931 | 0.0319 | 0.1178 | 0.0002 | 0.0706 | 0.1095 | 1.94E-07 | (*DRD1*) |
| Additive | 2 | rs6041532 | 20 | 12652435 | 0.2774 | 0.0018 | 0.1868 | 0.0374 | 0.2971 | 0.0163 | 0.4835 | 0.2387 | 1.61E-05 | - |
| Additive | 3 | rs9354118 | 6 | 95147902 | 0.064 | 0.0301 | 0.0847 | 0.0082 | 0.0772 | 0.0174 | 0.4835 | 0.07504 | 2.12E-05 | - |
| Additive | 4 | rs10170463 | 2 | 28773874 | -0.0821 | 0.0117 | -0.1091 | 0.0022 | -0.0743 | 0.0471 | - | -0.08345 | 2.97E-05 | *PLB1* |
| Additive | 5 | rs9342409 | 6 | 95098682 | 0.0624 | 0.0326 | 0.0845 | 0.0112 | 0.0737 | 0.0242 | - | 0.07408 | 3.31E-05 | - |
| Additive | 6 | rs35018407 | 17 | 62690181 | -0.1786 | 0.0449 | -0.1069 | 0.0312 | -0.1762 | 0.0043 | - | -0.1416 | 4.41E-05 | - |
| Additive | 7 | rs452325 | 8 | 88505366 | 0.0806 | 0.0126 | 0.0842 | 0.0224 | 0.0759 | 0.0203 | - | 0.07679 | 4.49E-05 | - |
| Additive | 8 | rs2676289 | 17 | 62705738 | -0.1797 | 0.0435 | -0.1114 | 0.0245 | -0.1727 | 0.0053 | 0.4835 | -0.1414 | 4.52E-05 | - |
| Additive | 9 | rs6504249 | 17 | 62676149 | -0.1786 | 0.0449 | -0.1069 | 0.0312 | -0.1743 | 0.0048 | - | -0.1405 | 5.04E-05 | - |
| Additive | 10 | rs4764074 | 12 | 14428118 | -0.1019 | 0.0096 | -0.0773 | 0.0293 | -0.0695 | 0.0469 | 0.6942 | -0.08307 | 5.39E-05 | - |
| Additive | 11 | rs9363197 | 6 | 95104559 | 0.064 | 0.0301 | 0.0738 | 0.0246 | 0.0737 | 0.0242 | - | 0.07127 | 6.17E-05 | - |
| Additive | 12 | rs375481 | 8 | 88490938 | 0.0814 | 0.0126 | 0.0887 | 0.0137 | 0.0711 | 0.03 | 0.5764 | 0.07512 | 6.36E-05 | - |
| Additive | 13 | exm2270886 | 8 | 88503249 | 0.0793 | 0.0143 | 0.0842 | 0.0224 | 0.07 | 0.0306 | - | 0.07426 | 7.09E-05 | - |
| Additive | 14 | rs391916 | 8 | 88512286 | 0.0835 | 0.012 | 0.0785 | 0.0358 | 0.0766 | 0.0251 | - | 0.07711 | 7.13E-05 | - |
| Additive | 15 | rs4806716 | 19 | 54639868 | -0.0667 | 0.0283 | -0.0814 | 0.0206 | -0.0783 | 0.0155 | 0.4835 | -0.07262 | 7.39E-05 | - |
| Additive | 16 | rs2759632 | 10 | 10218843 | -0.1186 | 0.0239 | -0.118 | 0.0426 | -0.1189 | 0.0142 | 0.4835 | -0.1163 | 0.000103 | - |
| Additive | 17 | rs463809 | 8 | 88513842 | 0.082 | 0.0133 | 0.0777 | 0.0364 | 0.0701 | 0.0379 | - | 0.07432 | 0.000111 | - |
| Additive | 18 | rs1016214 | 20 | 16992615 | 0.1575 | 0.0299 | 0.2567 | 0.0467 | 0.1343 | 0.0098 | 0.4835 | 0.1483 | 0.000166 | - |
| Additive | 19 | rs16978169 | 18 | 42361969 | -0.1468 | 0.0106 | -0.1063 | 0.0393 | -0.1052 | 0.0434 | 0.668 | -0.1116 | 0.000228 | *SETBP1* |
| Additive | 20 | rs7114479 | 11 | 2763228 | -0.1414 | 0.048 | -0.1399 | 0.0307 | -0.1018 | 0.0411 | 0.6571 | -0.1229 | 0.000275 | *KCNQ1* |
| Additive | 21 | rs652930 | 1 | 201628577 | 0.1122 | 0.03 | 0.1179 | 0.0452 | 0.2116 | 0.0159 | 0.4835 | 0.1172 | 0.000811 | *NAV1* |
| Dominant | 1 | rs375071 | 8 | 88532587 | -0.1435 | 0.0021 | -0.1759 | 0.0012 | -0.0961 | 0.0403 | - | -0.1363 | 8.07E-07 | - |
| Dominant | 2 | rs6502266 | 17 | 13395720 | 0.1549 | 0.0009 | 0.1337 | 0.0047 | 0.092 | 0.0435 | 0.5363 | 0.1223 | 2.6E-06 | - |
| Dominant | 3 | rs416011 | 8 | 88533806 | -0.1292 | 0.0067 | -0.1665 | 0.0025 | -0.0967 | 0.0462 | 0.5363 | -0.13 | 4.71E-06 | - |
| Dominant | 4 | rs17738087 | 15 | 26905021 | -0.1405 | 0.0084 | -0.1397 | 0.0087 | -0.1184 | 0.0112 | 0.414 | -0.1249 | 1.38E-05 | *GABRB3* |
| Dominant | 5 | rs751687 | 8 | 15608896 | 0.1396 | 0.0024 | 0.1118 | 0.0172 | 0.0947 | 0.0371 | 0.5363 | 0.1137 | 1.41E-05 | *TUSC3* |
| Dominant | 6 | rs17081058 | 13 | 25267734 | -0.12 | 0.0145 | -0.1101 | 0.0282 | -0.1053 | 0.0385 | 0.5363 | -0.118 | 3.02E-05 | *ATP12A* |
| Dominant | 7 | rs11842177 | 13 | 112821684 | -0.1053 | 0.0226 | -0.0981 | 0.0432 | -0.1241 | 0.0101 | 0.414 | -0.1116 | 3.47E-05 | - |
| Dominant | 8 | rs4580854 | 6 | 15025298 | -0.096 | 0.0419 | -0.1101 | 0.0184 | -0.1212 | 0.0078 | 0.4074 | -0.1082 | 3.75E-05 | - |
| Dominant | 9 | rs1195916 | 12 | 131503109 | 0.0989 | 0.0471 | 0.0974 | 0.0439 | 0.1437 | 0.0059 | 0.4074 | 0.1135 | 5.11E-05 | *GPR133* |
| Dominant | 10 | rs172399 | 7 | 9154302 | 0.1279 | 0.0086 | 0.108 | 0.026 | 0.0932 | 0.0393 | 0.5363 | 0.1079 | 5.5E-05 | - |
| Dominant | 11 | rs10956972 | 8 | 87768331 | 0.1262 | 0.0139 | 0.1015 | 0.0393 | 0.1381 | 0.0061 | - | 0.1122 | 8.32E-05 | - |
| Dominant | 12 | rs1982563 | 8 | 87776019 | 0.1262 | 0.0139 | 0.1015 | 0.0393 | 0.1381 | 0.0061 | 0.4074 | 0.1122 | 8.32E-05 | - |
| Dominant | 13 | rs5766289 | 22 | 45408177 | -0.1006 | 0.0318 | -0.102 | 0.0311 | -0.1198 | 0.0088 | 0.4074 | -0.104 | 8.45E-05 | - |
| Dominant | 14 | rs2143500 | 20 | 45253237 | 0.0956 | 0.0432 | 0.1196 | 0.0145 | 0.1006 | 0.0304 | 0.5363 | 0.1033 | 0.000122 | *SLC13A3* |
| Dominant | 15 | rs404469 | 8 | 88472924 | 0.1214 | 0.0102 | 0.0991 | 0.0473 | 0.1201 | 0.0194 | 0.4228 | 0.1077 | 0.000128 | - |
| Dominant | 16 | rs7592517 | 2 | 76777279 | 0.1543 | 0.0027 | 0.1014 | 0.0398 | 0.0988 | 0.0377 | - | 0.1014 | 0.000191 | - |
| Dominant | 17 | rs2139502 | 2 | 76786845 | 0.1543 | 0.0027 | 0.1014 | 0.0398 | 0.0988 | 0.0377 | 0.5363 | 0.1014 | 0.000191 | - |
| Dominant | 18 | rs1027804 | 8 | 18919857 | -0.1012 | 0.0386 | -0.0973 | 0.0484 | -0.126 | 0.0083 | 0.4074 | -0.1022 | 0.000215 | - |
| Dominant | 19 | rs6792514 | 3 | 42429817 | 0.1445 | 0.036 | 0.164 | 0.0264 | 0.1132 | 0.036 | 0.5363 | 0.1304 | 0.000286 | - |
| Dominant | 20 | exm-rs10873636 | 15 | 26888978 | -0.1245 | 0.0241 | -0.12 | 0.0278 | -0.0962 | 0.0431 | - | -0.1046 | 0.000331 | *GABRB3* |
| Dominant | 21 | rs10873636 | 15 | 26888978 | -0.1245 | 0.0241 | -0.12 | 0.0278 | -0.0962 | 0.0431 | - | -0.1046 | 0.000331 | *GABRB3* |
| Dominant | 22 | rs1863459 | 15 | 26892676 | -0.1245 | 0.0241 | -0.12 | 0.0278 | -0.0962 | 0.0431 | 0.5363 | -0.1046 | 0.000331 | *GABRB3* |
| Dominant | 23 | rs11945758 | 4 | 118667234 | 0.1178 | 0.0279 | 0.1096 | 0.0379 | 0.1144 | 0.0206 | 0.4228 | 0.1042 | 0.00036 | - |
| Dominant | 24 | rs6667463 | 1 | 175518442 | -0.1019 | 0.042 | -0.1442 | 0.0093 | -0.1 | 0.0464 | 0.5363 | -0.1019 | 0.000399 | *TNR* |
| Recessive | 1 | rs966775 | 5 | 174763322 | 0.2018 | 0.0104 | 0.195 | 0.02 | 0.1954 | 0.0009 | 0.3501 | 0.1982 | 7.66E-07 | (*DRD1*) |
| Recessive | 2 | rs6041532 | 20 | 12652435 | 0.5559 | 0.0016 | 0.3698 | 0.0381 | 0.6042 | 0.0143 | 0.385 | 0.4785 | 1.43E-05 | - |
| Recessive | 3 | rs9354118 | 6 | 95147902 | 0.1061 | 0.0361 | 0.1287 | 0.0225 | 0.1658 | 0.0017 | 0.3501 | 0.1306 | 1.51E-05 | - |
| Recessive | 4 | rs9342409 | 6 | 95098682 | 0.1014 | 0.0436 | 0.1317 | 0.0269 | 0.1658 | 0.0017 | - | 0.1309 | 1.86E-05 | - |
| Recessive | 5 | rs11160335 | 14 | 96867741 | -0.108 | 0.0456 | -0.1867 | 0.0013 | -0.1063 | 0.031 | 0.5398 | -0.1233 | 3.93E-05 | *AK7* |
| Recessive | 6 | rs4764074 | 12 | 14428118 | -0.1835 | 0.0147 | -0.1395 | 0.0363 | -0.1572 | 0.0157 | 0.385 | -0.1583 | 4.56E-05 | - |
| Recessive | 7 | rs10235250 | 7 | 153947649 | 0.1293 | 0.0466 | 0.2032 | 0.0139 | 0.1612 | 0.011 | 0.385 | 0.1555 | 7.6E-05 | *DPP6* |
| Recessive | 8 | rs35018407 | 17 | 62690181 | -0.3481 | 0.0494 | -0.193 | 0.0486 | -0.337 | 0.0062 | - | -0.2717 | 8.25E-05 | - |
| Recessive | 9 | rs2759632 | 10 | 10218843 | -0.2264 | 0.0288 | -0.2323 | 0.0424 | -0.2552 | 0.0079 | 0.385 | -0.2318 | 8.75E-05 | - |
| Recessive | 10 | rs6504249 | 17 | 62676149 | -0.3481 | 0.0494 | -0.193 | 0.0486 | -0.3341 | 0.0067 | - | -0.2698 | 9.21E-05 | - |
| Recessive | 10 | rs2676289 | 17 | 62705738 | -0.3481 | 0.0494 | -0.193 | 0.0486 | -0.3341 | 0.0067 | 0.385 | -0.2698 | 9.21E-05 | - |
| Recessive | 12 | rs375481 | 8 | 88490938 | 0.1323 | 0.0149 | 0.1636 | 0.013 | 0.1172 | 0.0413 | 0.6462 | 0.1289 | 9.76E-05 | - |
| Recessive | 13 | rs43211 | 19 | 54652203 | -0.1058 | 0.0416 | -0.1741 | 0.0093 | -0.1401 | 0.0156 | 0.385 | -0.1261 | 0.000103 | *CNOT3* |
| Recessive | 14 | rs12714409 | 2 | 596532 | 0.1209 | 0.0299 | 0.1252 | 0.0386 | 0.1419 | 0.0159 | 0.385 | 0.1257 | 0.000144 | - |
| Recessive | 15 | rs2146423 | 9 | 4657040 | 0.1567 | 0.0266 | 0.1518 | 0.036 | 0.2153 | 0.0055 | 0.385 | 0.1549 | 0.000169 | *C9orf68* |
| Recessive | 16 | rs452325 | 8 | 88505366 | 0.1205 | 0.0258 | 0.1386 | 0.0423 | 0.1304 | 0.0248 | - | 0.1251 | 0.000192 | - |
| Recessive | 17 | rs13325398 | 3 | 45161070 | 0.4936 | 0.0489 | 0.5272 | 0.0392 | 0.6042 | 0.0143 | 0.385 | 0.5311 | 0.000197 | *CDCP1* |
| Recessive | 18 | rs7190119 | 16 | 80926866 | 0.1329 | 0.0322 | 0.1355 | 0.0358 | 0.1047 | 0.0495 | 0.6462 | 0.1251 | 0.000203 | - |
| Recessive | 19 | exm2270886 | 8 | 88503249 | 0.1276 | 0.0171 | 0.1386 | 0.0423 | 0.1172 | 0.0413 | - | 0.122 | 0.000234 | - |

CHR, chromosome number; Position, chromosomal position (bp); *q*, *q* value for FDR correction of multiple comparison; Related gene, nearest gene from the SNP site.

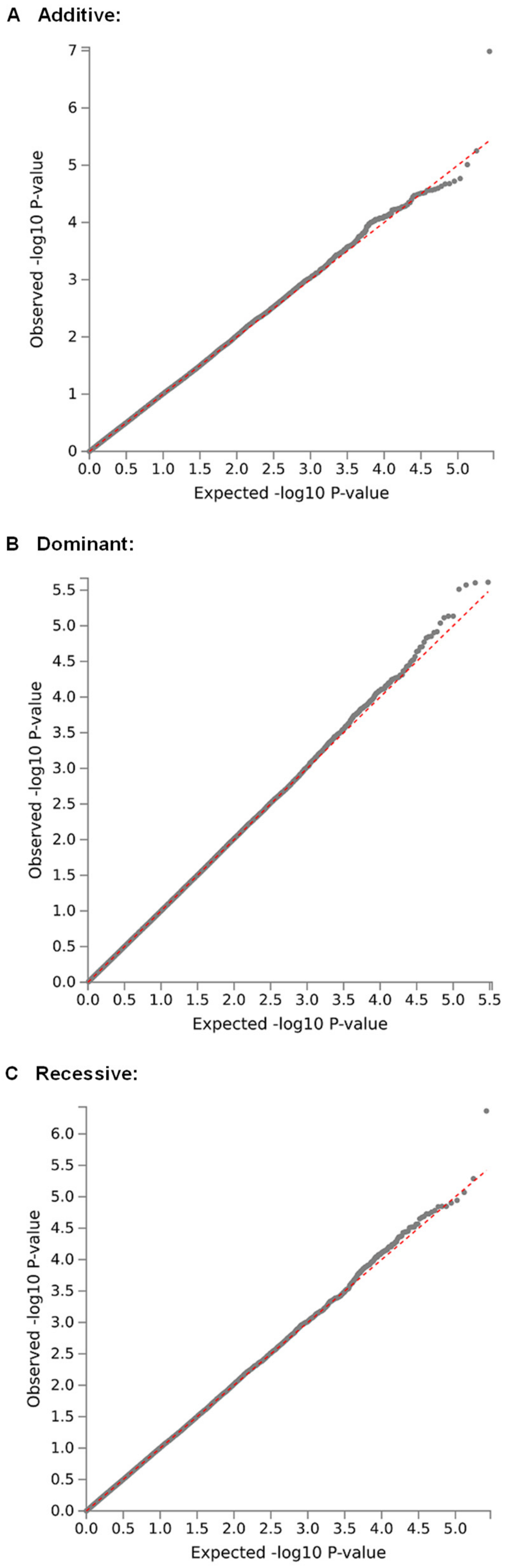


**Figure S1.** Log quantile-quantile (QQ) *p*-value plot as a result of the GWAS for the 0-6 h plasma MEC in combined samples. (**A**) Plot of the results from the additive model. (**B**) Plot of the results from the dominant model. (**C**) Plot of the results from the recessive model.

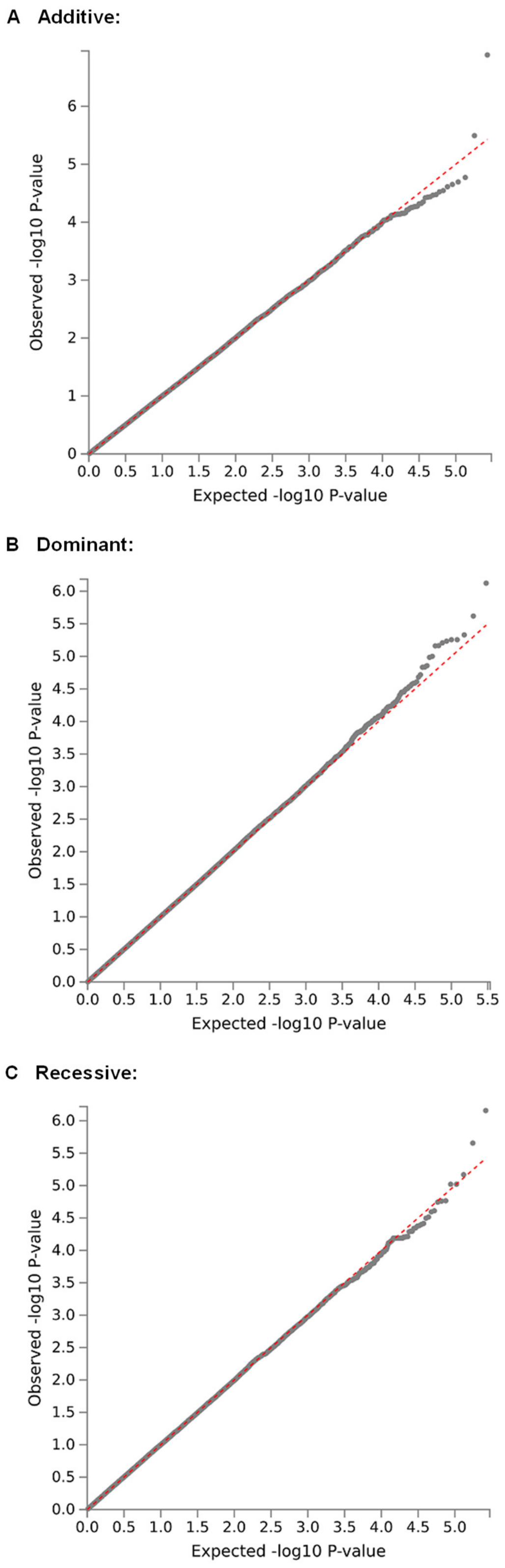


**Figure S2.** Log quantile-quantile (QQ) *p*-value plot as a result of the GWAS for the 0-12 h effect site MEC in combined samples. (**A**) Plot of results from the additive model. (**B**) Plot of results from the dominant model. (**C**) Plot of results from the recessive model.

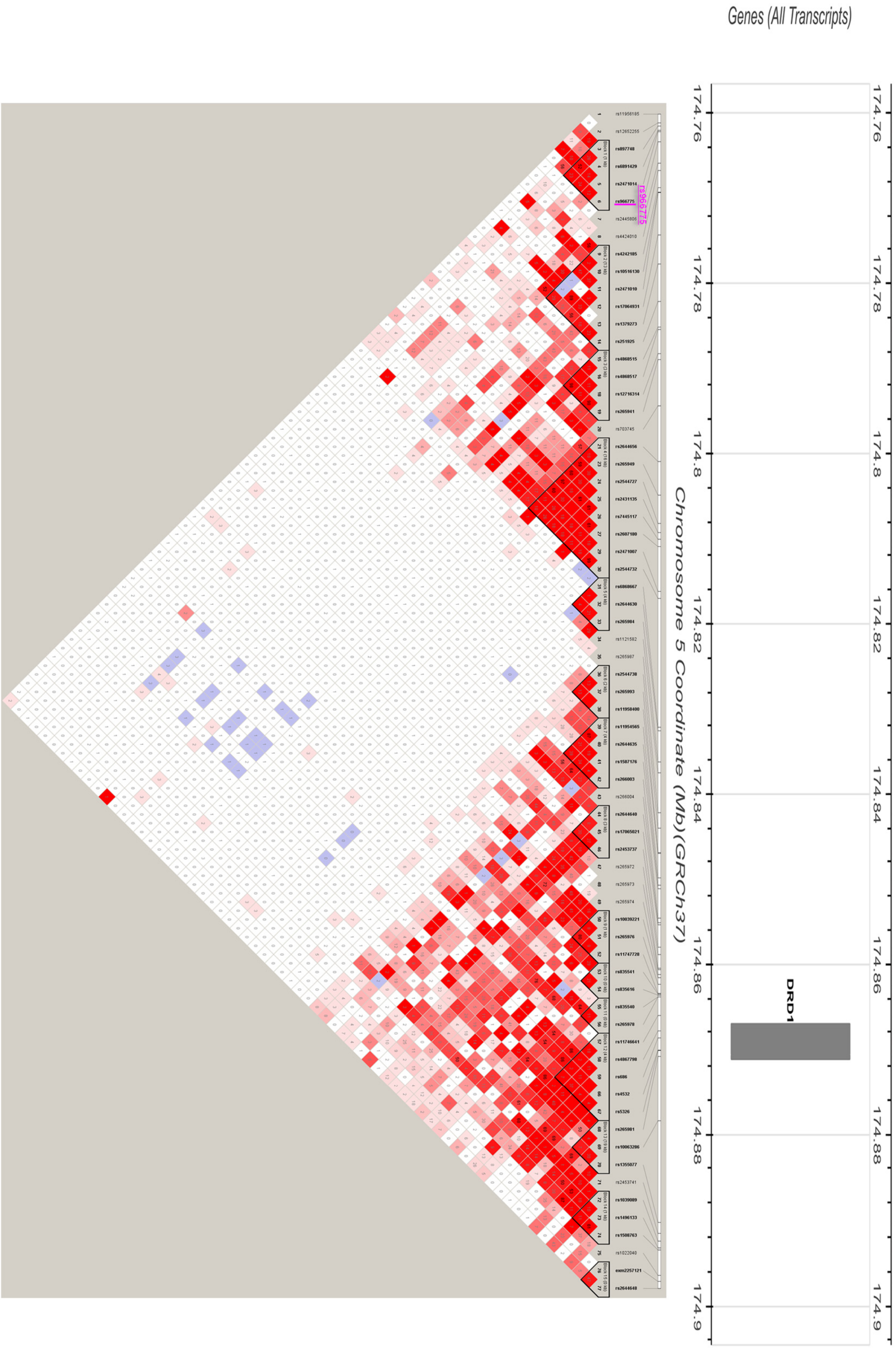


**Figure S3.** State of LD between SNPs in the genomic position from ~174 760 000 to ~174 900 000 on chromosome 5 (GRCh37) that includes both of the rs966775 SNP and *DRD1* gene and its flanking region. Genotype data for the 351 patient subjects that passed the quality control criteria were used in the LD analysis, and SNPs with a minor allele frequency ≥ 0.05 were selected in the genomic position. Numbers in squares in which two SNPs face each other represent the percentage of $r^2$ values that were calculated from genotype data of the SNPs. Blank squares represent $r^2 = 1$. The gray box above the LD plot represents a transcript of the *DRD1* gene. The rs966775 SNP is highlighted with the pink line.

**Table S4.** Top 20 candidate genes selected from gene-based analysis for the 0-12 h plasma MEC.

| Model | Rank | CHR | Gene start position | Gene stop position | Gene | nSNPs | Z Statistic | *p* | *p*[a] |
|---|---|---|---|---|---|---|---|---|---|
| Additive | 1 | 2 | 220378892 | 220403494 | *ASIC4* | 6 | 4.0611 | 0.000024419 | 0.422106834 |
| Additive | 2 | 9 | 132500610 | 132515326 | *PTGES* | 6 | 4.0031 | 0.000031262 | 0.540394932 |
| Additive | 3 | 2 | 220299568 | 220363009 | *SPEG* | 15 | 3.7604 | 0.000084831 | 1 |
| Additive | 4 | 2 | 20448452 | 20551995 | *PUM2* | 6 | 3.6547 | 0.00012876 | 1 |
| Additive | 5 | 11 | 67195931 | 67202872 | *RPS6KB2* | 2 | 3.5644 | 0.00018238 | 1 |
| Additive | 6 | 4 | 169418217 | 169849608 | *PALLD* | 119 | 3.3677 | 0.00037896 | 1 |
| Additive | 7 | X | 135295381 | 135338641 | *MAP7D3* | 6 | 3.3451 | 0.00041122 | 1 |
| Additive | 8 | 11 | 51515282 | 51516211 | *OR4C46* | 1 | 3.2728 | 0.0005324 | 1 |
| Additive | 9 | 11 | 51411378 | 51412448 | *OR4A5* | 2 | 3.2099 | 0.000664 | 1 |
| Additive | 10 | 12 | 122089024 | 122110537 | *MORN3* | 3 | 3.2092 | 0.00066543 | 1 |
| Additive | 11 | 2 | 178477720 | 178483694 | *TTC30A* | 6 | 3.1978 | 0.00069236 | 1 |
| Additive | 12 | 10 | 18240768 | 18332221 | *SLC39A12* | 37 | 3.1733 | 0.0007537 | 1 |
| Additive | 13 | 1 | 41157320 | 41237275 | *NFYC* | 5 | 3.0441 | 0.001167 | 1 |
| Additive | 14 | 1 | 235490665 | 235507847 | *GGPS1* | 4 | 3.0309 | 0.001219 | 1 |
| Additive | 15 | 2 | 204259068 | 204400133 | *RAPH1* | 9 | 3.0173 | 0.0012753 | 1 |
| Additive | 16 | 17 | 42325753 | 42345509 | *SLC4A1* | 10 | 2.9995 | 0.0013523 | 1 |
| Additive | 17 | 13 | 25254549 | 25285921 | *ATP12A* | 14 | 2.9816 | 0.0014336 | 1 |
| Additive | 18 | 2 | 242673994 | 242708231 | *D2HGDH* | 2 | 2.9715 | 0.0014818 | 1 |
| Additive | 19 | 2 | 204192942 | 204312446 | *ABI2* | 6 | 2.9521 | 0.0015781 | 1 |
| Additive | 20 | 7 | 44836279 | 44864163 | *PPIA* | 3 | 2.9399 | 0.0016418 | 1 |
| Dominant | 1 | 13 | 44947801 | 44971850 | *SERP2* | 5 | 4.686 | 1.3932E-06 | 0.024381* |
| Dominant | 2 | 4 | 169418217 | 169849608 | *PALLD* | 134 | 3.8521 | 0.000058545 | 1 |
| Dominant | 3 | 13 | 25254549 | 25285921 | *ATP12A* | 14 | 3.718 | 0.00010038 | 1 |
| Dominant | 4 | 20 | 45186463 | 45304714 | *SLC13A3* | 79 | 3.6989 | 0.00010825 | 1 |
| Dominant | 5 | 14 | 57936019 | 57960585 | *C14orf105* | 7 | 3.6821 | 0.00011564 | 1 |
| Dominant | 6 | 17 | 4574679 | 4607632 | *PELP1* | 4 | 3.57 | 0.00017849 | 1 |
| Dominant | 7 | 2 | 218148742 | 218621316 | *DIRC3* | 104 | 3.3557 | 0.00039586 | 1 |
| Dominant | 8 | 10 | 22823778 | 23003484 | *PIP4K2A* | 47 | 3.3258 | 0.00044086 | 1 |
| Dominant | 9 | 10 | 5435061 | 5446793 | *TUBAL3* | 7 | 3.3245 | 0.00044285 | 1 |
| Dominant | 10 | 7 | 1509913 | 1545489 | *INTS1* | 5 | 3.3054 | 0.00047418 | 1 |
| Dominant | 11 | 1 | 41157320 | 41237275 | *NFYC* | 5 | 3.2557 | 0.00056559 | 1 |
| Dominant | 12 | 14 | 104552016 | 104579098 | *ASPG* | 6 | 3.241 | 0.00059556 | 1 |
| Dominant | 13 | 11 | 63580860 | 63595190 | *C11orf84* | 5 | 3.2264 | 0.00062679 | 1 |
| Dominant | 14 | 5 | 128430444 | 128449721 | *ISOC1* | 9 | 3.2037 | 0.00067833 | 1 |
| Dominant | 15 | 13 | 45007655 | 45151283 | *TSC22D1* | 13 | 3.1633 | 0.00077992 | 1 |
| Dominant | 16 | 17 | 5402747 | 5522744 | *NLRP1* | 37 | 3.1574 | 0.00079602 | 1 |
| Dominant | 17 | 8 | 145691426 | 145699585 | *KIFC2* | 2 | 3.1443 | 0.00083242 | 1 |
| Dominant | 18 | 9 | 22002902 | 22009362 | *CDKN2B* | 3 | 3.1212 | 0.00090071 | 1 |
| Dominant | 19 | 8 | 145106167 | 145118735 | *OPLAH* | 8 | 3.1127 | 0.00092685 | 1 |
| Dominant | 20 | 1 | 33979609 | 34631443 | *CSMD2* | 246 | 3.0998 | 0.00096815 | 1 |
| Recessive | 1 | 9 | 132500610 | 132515326 | *PTGES* | 6 | 4.1021 | 0.000020475 | 0.342157725 |
| Recessive | 2 | 2 | 220378892 | 220403494 | *ASIC4* | 6 | 4.0525 | 0.00002534 | 0.42345674 |
| Recessive | 3 | 11 | 51515282 | 51516211 | *OR4C46* | 1 | 3.5392 | 0.0002007 | 1 |
| Recessive | 4 | 11 | 67195931 | 67202872 | *RPS6KB2* | 2 | 3.4952 | 0.00023683 | 1 |
| Recessive | 5 | 11 | 51411378 | 51412448 | *OR4A5* | 2 | 3.3992 | 0.00033794 | 1 |
| Recessive | 6 | 2 | 220299568 | 220363009 | *SPEG* | 15 | 3.368 | 0.00037858 | 1 |
| Recessive | 7 | 2 | 20448452 | 20551995 | *PUM2* | 6 | 3.3558 | 0.00039575 | 1 |
| Recessive | 8 | 2 | 178477720 | 178483694 | *TTC30A* | 6 | 3.2888 | 0.000503 | 1 |
| Recessive | 9 | 12 | 122089024 | 122110537 | *MORN3* | 3 | 3.2397 | 0.00059823 | 1 |
| Recessive | 10 | 7 | 44836279 | 44864163 | *PPIA* | 3 | 3.1541 | 0.00080508 | 1 |
| Recessive | 11 | 11 | 55563032 | 55563976 | *OR5D14* | 2 | 3.0801 | 0.0010346 | 1 |
| Recessive | 12 | 16 | 88519725 | 88603424 | *ZFPM1* | 10 | 3.0574 | 0.0011164 | 1 |
| Recessive | 13 | 1 | 44398992 | 44402913 | *ARTN* | 2 | 3.0501 | 0.0011436 | 1 |
| Recessive | 14 | 2 | 204259068 | 204400133 | *RAPH1* | 9 | 3.0442 | 0.0011666 | 1 |
| Recessive | 15 | 1 | 45240923 | 45244451 | *RPS8* | 1 | 2.9911 | 0.00139 | 1 |
| Recessive | 16 | 2 | 204192942 | 204312446 | *ABI2* | 6 | 2.9847 | 0.0014192 | 1 |
| Recessive | 17 | 2 | 242673994 | 242708231 | *D2HGDH* | 2 | 2.9638 | 0.0015193 | 1 |
| Recessive | 18 | 17 | 26975374 | 26989207 | *SDF2* | 1 | 2.9125 | 0.001793 | 1 |
| Recessive | 19 | 1 | 44440159 | 44443967 | *ATP6V0B* | 3 | 2.9031 | 0.0018473 | 1 |
| Recessive | 20 | 12 | 54104903 | 54121529 | *CALCOCO1* | 9 | 2.8837 | 0.0019649 | 1 |

Model, the genetic model in which candidate genes were selected by analysis; CHR, chromosome number; nSNPs, number of SNPs annotated to the gene; Z Statistic, gene-based test statistic; $p^a$, adjusted $p$ value for multiple testing. *Significant association after Bonferroni correction.

**Table S5.** Top 20 candidate genes selected from gene-based analysis for the 0-6 h effect site MEC.

| Model | Rank | CHR | Gene start position | Gene stop position | Gene | nSNPs | Z Statistic | *p* | *p*[a] |
|---|---|---|---|---|---|---|---|---|---|
| Additive | 1 | X | 135295381 | 135338641 | *MAP7D3* | 6 | 3.9881 | 0.000033301 | 0.575641086 |
| Additive | 2 | 2 | 20448452 | 20551995 | *PUM2* | 6 | 3.6488 | 0.00013172 | 1 |
| Additive | 3 | 2 | 220378892 | 220403494 | *ASIC4* | 6 | 3.6466 | 0.00013288 | 1 |
| Additive | 4 | 2 | 28680012 | 28866654 | *PLB1* | 66 | 3.5024 | 0.00023058 | 1 |
| Additive | 5 | 2 | 220299568 | 220363009 | *SPEG* | 15 | 3.4767 | 0.00025377 | 1 |
| Additive | 6 | 9 | 132500610 | 132515326 | *PTGES* | 6 | 3.4024 | 0.00033397 | 1 |
| Additive | 7 | 4 | 169418217 | 169849608 | *PALLD* | 119 | 3.3236 | 0.00044438 | 1 |
| Additive | 8 | 11 | 67195931 | 67202872 | *RPS6KB2* | 2 | 3.2845 | 0.00051075 | 1 |
| Additive | 9 | 12 | 122089024 | 122110537 | *MORN3* | 3 | 3.2289 | 0.00062137 | 1 |
| Additive | 10 | 12 | 12813825 | 12849141 | *GPR19* | 11 | 3.1949 | 0.0006993 | 1 |
| Additive | 11 | 1 | 235490665 | 235507847 | *GGPS1* | 4 | 3.1751 | 0.00074887 | 1 |
| Additive | 12 | 11 | 51515282 | 51516211 | *OR4C46* | 1 | 3.1434 | 0.0008349 | 1 |
| Additive | 13 | 11 | 51411378 | 51412448 | *OR4A5* | 2 | 3.1194 | 0.00090608 | 1 |
| Additive | 14 | 17 | 56597611 | 56618179 | *44808* | 5 | 3.0893 | 0.0010032 | 1 |
| Additive | 15 | 1 | 41157320 | 41237275 | *NFYC* | 5 | 3.087 | 0.0010109 | 1 |
| Additive | 16 | 2 | 20400558 | 20425194 | *SDC1* | 2 | 3.0032 | 0.0013358 | 1 |
| Additive | 17 | 18 | 24432002 | 24445782 | *AQP4* | 2 | 2.9345 | 0.0016706 | 1 |
| Additive | 18 | 17 | 42325753 | 42345509 | *SLC4A1* | 10 | 2.9106 | 0.0018036 | 1 |
| Additive | 19 | 4 | 178163693 | 178169927 | *RP11-487E13.1* | 3 | 2.8614 | 0.002109 | 1 |
| Additive | 20 | 2 | 44589089 | 44999731 | *CAMKMT* | 54 | 2.8579 | 0.002132 | 1 |
| Dominant | 1 | 13 | 44947801 | 44971850 | *SERP2* | 5 | 4.5266 | 2.9972E-06 | 0.052451 |
| Dominant | 2 | 4 | 169418217 | 169849608 | *PALLD* | 134 | 3.962 | 0.00003716 | 0.6503 |
| Dominant | 3 | 20 | 45186463 | 45304714 | *SLC13A3* | 79 | 3.67 | 0.00012126 | 1 |
| Dominant | 4 | 17 | 5402747 | 5522744 | *NLRP1* | 37 | 3.6646 | 0.00012387 | 1 |
| Dominant | 5 | 13 | 25254549 | 25285921 | *ATP12A* | 14 | 3.6167 | 0.00014922 | 1 |
| Dominant | 6 | 1 | 41157320 | 41237275 | *NFYC* | 5 | 3.5564 | 0.00018796 | 1 |
| Dominant | 7 | 17 | 4574679 | 4607632 | *PELP1* | 4 | 3.518 | 0.00021738 | 1 |
| Dominant | 8 | 10 | 22823778 | 23003484 | *PIP4K2A* | 47 | 3.2921 | 0.00049725 | 1 |
| Dominant | 9 | 5 | 140579183 | 140582618 | *PCDHB11* | 1 | 3.2904 | 0.0005002 | 1 |
| Dominant | 10 | 8 | 87878670 | 88627447 | *CNBD1* | 106 | 3.2477 | 0.00058163 | 1 |
| Dominant | 11 | 19 | 8271620 | 8327305 | *CERS4* | 23 | 3.2301 | 0.00061879 | 1 |
| Dominant | 12 | 14 | 57936019 | 57960585 | *C14orf105* | 7 | 3.1922 | 0.00070587 | 1 |
| Dominant | 13 | 7 | 1509913 | 1545489 | *INTS1* | 5 | 3.1899 | 0.00071154 | 1 |
| Dominant | 14 | 14 | 104552016 | 104579098 | *ASPG* | 6 | 3.1778 | 0.00074194 | 1 |
| Dominant | 15 | 10 | 5435061 | 5446793 | *TUBAL3* | 7 | 3.1663 | 0.00077192 | 1 |
| Dominant | 16 | 5 | 140557371 | 140560081 | *PCDHB8* | 4 | 3.1339 | 0.00086238 | 1 |
| Dominant | 17 | 1 | 153389000 | 153395701 | *S100A7A* | 1 | 3.129 | 0.0008771 | 1 |
| Dominant | 18 | 13 | 112240548 | 112324955 | *RP11-65D24.2* | 22 | 3.0572 | 0.0011169 | 1 |
| Dominant | 19 | 13 | 45007655 | 45151283 | *TSC22D1* | 13 | 3.0458 | 0.0011601 | 1 |
| Dominant | 20 | 17 | 4613784 | 4624794 | *ARRB2* | 1 | 3.02 | 0.001264 | 1 |
| Recessive | 1 | 2 | 220378892 | 220403494 | *ASIC4* | 6 | 3.6931 | 0.00011077 | 1 |
| Recessive | 2 | 11 | 51515282 | 51516211 | *OR4C46* | 1 | 3.5233 | 0.0002131 | 1 |
| Recessive | 3 | 9 | 132500610 | 132515326 | *PTGES* | 6 | 3.5098 | 0.00022422 | 1 |
| Recessive | 4 | 2 | 20448452 | 20551995 | *PUM2* | 6 | 3.3788 | 0.00036404 | 1 |
| Recessive | 5 | 11 | 51411378 | 51412448 | *OR4A5* | 2 | 3.3257 | 0.00044102 | 1 |
| Recessive | 6 | 12 | 122089024 | 122110537 | *MORN3* | 3 | 3.2981 | 0.00048674 | 1 |
| Recessive | 7 | 11 | 67195931 | 67202872 | *RPS6KB2* | 2 | 3.2338 | 0.00061069 | 1 |
| Recessive | 8 | 2 | 220299568 | 220363009 | *SPEG* | 15 | 3.1592 | 0.00079097 | 1 |
| Recessive | 9 | 11 | 55563032 | 55563976 | *OR5D14* | 2 | 3.0825 | 0.0010265 | 1 |
| Recessive | 10 | 12 | 12813825 | 12849141 | *GPR19* | 11 | 3.039 | 0.001187 | 1 |
| Recessive | 11 | 17 | 56597611 | 56618179 | *44808* | 5 | 3.002 | 0.001341 | 1 |
| Recessive | 12 | 11 | 60197062 | 60222687 | *MS4A5* | 8 | 2.9601 | 0.0015378 | 1 |
| Recessive | 13 | 18 | 43405477 | 43424045 | *SIGLEC15* | 6 | 2.9114 | 0.0017993 | 1 |
| Recessive | 14 | 13 | 113812968 | 113826694 | *PROZ* | 1 | 2.8852 | 0.001956 | 1 |
| Recessive | 15 | 2 | 178477720 | 178483694 | *TTC30A* | 6 | 2.8757 | 0.002016 | 1 |
| Recessive | 16 | 4 | 156129781 | 156138230 | *NPY2R* | 2 | 2.8689 | 0.0020596 | 1 |
| Recessive | 17 | 1 | 235490665 | 235507847 | *GGPS1* | 4 | 2.8467 | 0.0022084 | 1 |
| Recessive | 18 | 17 | 41717756 | 41739322 | *MEOX1* | 5 | 2.8445 | 0.0022238 | 1 |
| Recessive | 19 | 19 | 39926796 | 39967310 | *SUPT5H* | 10 | 2.8252 | 0.0023626 | 1 |
| Recessive | 20 | 17 | 26975374 | 26989207 | *SDF2* | 1 | 2.8199 | 0.002402 | 1 |

Model, the genetic model in which candidate genes were selected by analysis; CHR, chromosome number; nSNPs, number of SNPs annotated to the gene; Z Statistic, gene-based test statistic; $p^a$, adjusted *p* value for multiple testing

**Table S6.** Top 20 candidate gene sets selected from gene-set analysis for the 0-6 h plasma MEC.

| Model | Rank | Gene set name | nGenes | Beta | SE | *p* | *p* [a] |
|---|---|---|---|---|---|---|---|
| Additive | 1 | go_paracrine_signaling | 7 | 1.3774 | 0.28547 | 7.0619E-07 | 0.010933940* |
| Additive | 2 | reactome_free_fatty_acid_receptors | 3 | 1.9291 | 0.40432 | 9.237E-07 | 0.014301647* |
| Additive | 3 | go_taste_receptor_activity | 20 | 1.1062 | 0.23947 | 1.9399E-06 | 0.030035472* |
| Additive | 4 | go_sensory_perception_of_taste | 53 | 0.56456 | 0.12766 | 4.9162E-06 | 0.076117525 |
| Additive | 5 | go_small_gtpase_mediated_signal_transduction | 522 | 0.15732 | 0.036233 | 7.1045E-06 | 0.109998974 |
| Additive | 6 | go_negative_regulation_of_epidermal_cell_differentiation | 11 | 0.97596 | 0.22563 | 7.6576E-06 | 0.118562621 |
| Additive | 7 | go_ccr2_chemokine_receptor_binding | 3 | 2.1321 | 0.49351 | 7.8404E-06 | 0.121392913 |
| Additive | 8 | go_negative_regulation_of_keratinocyte_differentiation | 7 | 1.129 | 0.26352 | 9.2146E-06 | 0.142669652 |
| Additive | 9 | go_detection_of_chemical_stimulus_involved_in_sensory_perception_of_taste | 34 | 0.69397 | 0.16211 | 9.3621E-06 | 0.144953394 |
| Additive | 10 | go_calcineurin_mediated_signaling | 40 | 0.56844 | 0.13869 | 0.000020881 | 0.323300523 |
| Additive | 11 | sotiriou_breast_cancer_grade_1_vs_3_dn | 48 | 0.42602 | 0.10496 | 0.000024777 | 0.383622291 |
| Additive | 12 | go_atp_dependent_3_5_dna_helicase_activity | 10 | 0.8431 | 0.21044 | 0.000030961 | 0.479369163 |
| Additive | 13 | go_negative_regulation_of_epidermis_development | 14 | 0.82576 | 0.2079 | 0.00003581 | 0.55444623 |
| Additive | 14 | go_regulation_of_isomerase_activity | 7 | 0.92378 | 0.24025 | 0.000060488 | 0.936535704 |
| Additive | 15 | go_3_5_rna_helicase_activity | 19 | 0.59361 | 0.16236 | 0.00012839 | 1 |
| Additive | 16 | go_positive_regulation_of_posttranscriptional_gene_silencing | 22 | 0.61362 | 0.17064 | 0.00016207 | 1 |
| Additive | 17 | go_sensory_perception_of_chemical_stimulus | 323 | 0.20859 | 0.058055 | 0.000164 | 1 |
| Additive | 18 | go_regulation_of_calcineurin_mediated_signaling | 30 | 0.57295 | 0.16145 | 0.00019401 | 1 |
| Additive | 19 | go_inositol_phosphate_mediated_signaling | 49 | 0.45093 | 0.1273 | 0.0001988 | 1 |
| Additive | 20 | reactome_signaling_by_fgfr1_in_disease | 32 | 0.51868 | 0.14667 | 0.00020336 | 1 |
| Dominant | 1 | dazard_uv_response_cluster_g5 | 9 | 0.84459 | 0.21385 | 0.00003932 | 0.60871292 |
| Dominant | 2 | go_cluster_of_actin_based_cell_projections | 140 | 0.25248 | 0.070729 | 0.00017922 | 1 |
| Dominant | 3 | go_activating_transcription_factor_binding | 68 | 0.36003 | 0.10452 | 0.00028678 | 1 |
| Dominant | 4 | go_tooth_mineralization | 21 | 0.62407 | 0.18816 | 0.00045641 | 1 |
| Dominant | 5 | go_enamel_mineralization | 16 | 0.74794 | 0.22832 | 0.00052776 | 1 |
| Dominant | 6 | reactome_foxo_mediated_transcription_of_cell_death_genes | 15 | 0.72354 | 0.22599 | 0.00068441 | 1 |
| Dominant | 7 | go_substantia_nigra_development | 38 | 0.4184 | 0.13092 | 0.00069845 | 1 |
| Dominant | 8 | weston_vegfa_targets | 89 | 0.30319 | 0.096673 | 0.00085703 | 1 |
| Dominant | 9 | go_macrophage_colony_stimulating_factor_production | 5 | 1.2335 | 0.3937 | 0.00086653 | 1 |
| Dominant | 10 | hofmann_myelodysplastic_syndrom_high_risk_up | 8 | 0.70209 | 0.2245 | 0.00088347 | 1 |
| Dominant | 11 | go_dna_helicase_activity | 46 | 0.31476 | 0.10082 | 0.00089999 | 1 |
| Dominant | 12 | go_gator2_complex | 10 | 0.7784 | 0.25114 | 0.00097113 | 1 |
| Dominant | 13 | go_negative_regulation_of_osteoclast_differentiation | 24 | 0.54907 | 0.17723 | 0.00097575 | 1 |
| Dominant | 14 | reactome_sumoylation_of_transcription_factors | 19 | 0.58459 | 0.19401 | 0.0012944 | 1 |
| Dominant | 15 | go_regulation_of_response_to_oxidative_stress | 71 | 0.29526 | 0.098444 | 0.0013554 | 1 |
| Dominant | 16 | go_trna_pseudouridine_synthesis | 5 | 1.2225 | 0.41034 | 0.0014473 | 1 |
| Dominant | 17 | go_tendon_development | 3 | 1.7285 | 0.5816 | 0.0014816 | 1 |
| Dominant | 18 | abdulrahman_kidney_cancer_vhl_dn | 13 | 0.74252 | 0.2518 | 0.0015971 | 1 |
| Dominant | 19 | lei_hoxc8_targets_up | 10 | 0.67309 | 0.22904 | 0.0016498 | 1 |
| Dominant | 20 | go_ciliary_base | 34 | 0.38905 | 0.13301 | 0.001725 | 1 |
| Recessive | 1 | go_negative_regulation_of_epidermal_cell_differentiation | 11 | 1.0601 | 0.22399 | 1.1166E-06 | 0.017281618* |
| Recessive | 2 | go_paracrine_signaling | 7 | 1.3272 | 0.28343 | 1.4279E-06 | 0.022099608* |
| Recessive | 3 | go_negative_regulation_of_epidermis_development | 14 | 0.95001 | 0.20637 | 2.0957E-06 | 0.032435149* |
| Recessive | 4 | go_negative_regulation_of_keratinocyte_differentiation | 7 | 1.1871 | 0.26161 | 2.8685E-06 | 0.044395775* |
| Recessive | 5 | go_small_gtpase_mediated_signal_transduction | 495 | 0.15539 | 0.036744 | 0.000011798 | 0.182597646 |
| Recessive | 6 | shin_b_cell_lymphoma_cluster_6 | 6 | 1.2233 | 0.29029 | 0.000012601 | 0.195025677 |
| Recessive | 7 | go_ccr2_chemokine_receptor_binding | 3 | 2.0377 | 0.48998 | 0.000016092 | 0.249055884 |
| Recessive | 8 | kegg_peroxisome | 72 | 0.33622 | 0.08787 | 0.00006528 | 1 |
| Recessive | 9 | wakabayashi_adipogenesis_pparg_bound_36hr | 28 | 0.63888 | 0.16867 | 0.000076304 | 1 |
| Recessive | 10 | reactome_activated_ntrk2_signals_through_frs2_and_frs3 | 7 | 1.1939 | 0.3159 | 0.000078879 | 1 |
| Recessive | 11 | reactome_free_fatty_acid_receptors | 3 | 1.5075 | 0.40153 | 0.000087175 | 1 |
| Recessive | 12 | go_negative_regulation_of_epithelial_cell_differentiation | 33 | 0.53166 | 0.14503 | 0.00012362 | 1 |
| Recessive | 13 | lee_liver_cancer_acox1_up | 55 | 0.41931 | 0.11471 | 0.00012879 | 1 |
| Recessive | 14 | go_protein_glycosylation_in_golgi | 4 | 1.284 | 0.35229 | 0.00013433 | 1 |
| Recessive | 15 | reactome_signaling_by_fgfr1_in_disease | 32 | 0.51802 | 0.14562 | 0.00018782 | 1 |
| Recessive | 16 | reactome_activated_ntrk2_signals_through_ras | 6 | 1.2221 | 0.34445 | 0.00019461 | 1 |
| Recessive | 17 | go_taste_receptor_activity | 20 | 0.84212 | 0.23782 | 0.00019992 | 1 |
| Recessive | 18 | hoffman_clock_targets_up | 6 | 1.0087 | 0.28603 | 0.00021123 | 1 |
| Recessive | 19 | go_nuclear_envelope_lumen | 9 | 0.98863 | 0.28046 | 0.0002123 | 1 |
| Recessive | 20 | reactome_signaling_by_fgfr_in_disease | 53 | 0.4032 | 0.11464 | 0.00021869 | 1 |

Model, the genetic model in which candidate gene sets were selected by analysis; nGenes, number of genes in the data that are in the gene set; Beta, regression coefficient of the gene set; SE, standard error of the regression coefficient; $p^a$, adjusted *p* value for multiple testing. *Significant association after conservative Bonferroni correction.

**Table S7.** Top 20 candidate gene sets selected from gene-set analysis for the 0-12 h plasma MEC.

| Model | Rank | Gene set name | nGenes | Beta | SE | *p* | *p* [a] |
|---|---|---|---|---|---|---|---|
| Additive | 1 | sotiriou_breast_cancer_grade_1_vs_3_dn | 48 | 0.47129 | 0.10425 | 3.1035E-06 | 0.048051491* |
| Additive | 2 | pid_shp2_pathway | 53 | 0.50344 | 0.11691 | 8.3595E-06 | 0.129430139 |
| Additive | 3 | go_ccr2_chemokine_receptor_binding | 3 | 2.1032 | 0.49022 | 8.9734E-06 | 0.138935152 |
| Additive | 4 | go_small_gtpase_mediated_signal_transduction | 522 | 0.14564 | 0.035994 | 0.000026159 | 0.405019797 |
| Additive | 5 | go_calcineurin_mediated_signaling | 40 | 0.51515 | 0.13778 | 0.000092704 | 1 |
| Additive | 6 | go_positive_regulation_of_superoxide_anion_generation | 16 | 0.6591 | 0.18398 | 0.0001707 | 1 |
| Additive | 7 | go_intrinsic_apoptotic_signaling_pathway_in_response_to_oxidative_stress | 36 | 0.48547 | 0.13553 | 0.00017101 | 1 |
| Additive | 8 | go_response_to_interleukin_7 | 27 | 0.50699 | 0.14311 | 0.00019851 | 1 |
| Additive | 9 | holleman_asparaginase_resistance_all_dn | 22 | 0.6056 | 0.17213 | 0.00021775 | 1 |
| Additive | 10 | go_paracrine_signaling | 7 | 0.99091 | 0.28367 | 0.00023932 | 1 |
| Additive | 11 | go_regulation_of_rna_polymerase_ii_transcriptional_preinitiation_complex_assembly | 14 | 0.80448 | 0.23304 | 0.00027879 | 1 |
| Additive | 12 | go_regulation_of_isomerase_activity | 7 | 0.80439 | 0.23867 | 0.00037637 | 1 |
| Additive | 13 | go_regulation_of_calcineurin_mediated_signaling | 30 | 0.54021 | 0.16038 | 0.00037896 | 1 |
| Additive | 14 | go_positive_regulation_of_posttranscriptional_gene_silencing | 22 | 0.56239 | 0.16951 | 0.00045489 | 1 |
| Additive | 15 | kegg_amyotrophic_lateral_sclerosis_als | 48 | 0.38829 | 0.11735 | 0.00046941 | 1 |
| Additive | 16 | reactome_activated_ntrk2_signals_through_frs2_and_frs3 | 7 | 1.0441 | 0.3161 | 0.00047939 | 1 |
| Additive | 17 | go_regulation_of_collateral_sprouting | 20 | 0.69471 | 0.21065 | 0.00048799 | 1 |
| Additive | 18 | riggins_tamoxifen_resistance_dn | 204 | 0.19223 | 0.058294 | 0.00048853 | 1 |
| Additive | 19 | gazda_diamond_blackfan_anemia_progenitor_dn | 55 | 0.32307 | 0.098863 | 0.00054304 | 1 |
| Additive | 20 | azare_stat3_targets | 22 | 0.52729 | 0.16137 | 0.00054335 | 1 |
| Dominant | 1 | go_cluster_of_actin_based_cell_projections | 140 | 0.26218 | 0.070408 | 0.000098502 | 1 |
| Dominant | 2 | go_tooth_mineralization | 21 | 0.68795 | 0.1873 | 0.00012022 | 1 |
| Dominant | 3 | hofmann_myelodysplastic_syndrom_high_risk_up | 8 | 0.79143 | 0.22347 | 0.00019942 | 1 |
| Dominant | 4 | go_atp_binding_cassette_abc_transporter_complex | 7 | 1.1655 | 0.33276 | 0.00023093 | 1 |
| Dominant | 5 | go_enamel_mineralization | 16 | 0.79589 | 0.22728 | 0.00023165 | 1 |
| Dominant | 6 | reactome_clec7a_dectin_1_induces_nfat_activation | 11 | 0.95364 | 0.27408 | 0.00025185 | 1 |
| Dominant | 7 | go_activating_transcription_factor_binding | 68 | 0.35933 | 0.10405 | 0.00027748 | 1 |
| Dominant | 8 | go_gator2_complex | 10 | 0.82482 | 0.25 | 0.00048577 | 1 |
| Dominant | 9 | go_biomineral_tissue_development | 138 | 0.25334 | 0.077146 | 0.00051293 | 1 |
| Dominant | 10 | dazard_uv_response_cluster_g5 | 9 | 0.69471 | 0.21291 | 0.00055259 | 1 |
| Dominant | 11 | go_dna_helicase_activity | 46 | 0.31996 | 0.10037 | 0.00071797 | 1 |
| Dominant | 12 | go_bone_mineralization | 97 | 0.28535 | 0.091047 | 0.00086343 | 1 |
| Dominant | 13 | zheng_foxp3_targets_in_t_lymphocyte_dn | 37 | 0.43225 | 0.13932 | 0.000961 | 1 |
| Dominant | 14 | go_regulation_of_response_to_oxidative_stress | 71 | 0.30355 | 0.097999 | 0.00097734 | 1 |
| Dominant | 15 | go_corpus_callosum_morphogenesis | 6 | 1.0139 | 0.33154 | 0.0011154 | 1 |
| Dominant | 16 | go_seh1_associated_complex | 13 | 0.66562 | 0.22177 | 0.0013455 | 1 |
| Dominant | 17 | go_substantia_nigra_development | 38 | 0.38979 | 0.13033 | 0.0013938 | 1 |
| Dominant | 18 | lin_melanoma_copy_number_dn | 41 | 0.40422 | 0.13521 | 0.0013986 | 1 |
| Dominant | 19 | go_negative_regulation_of_osteoclast_differentiation | 24 | 0.52477 | 0.17644 | 0.0014706 | 1 |
| Dominant | 20 | go_response_to_extracellular_stimulus | 465 | 0.11572 | 0.039035 | 0.0015173 | 1 |
| Recessive | 1 | pid_shp2_pathway | 52 | 0.52819 | 0.12027 | 5.6651E-06 | 0.087678753 |
| Recessive | 2 | go_small_gtpase_mediated_signal_transduction | 495 | 0.15847 | 0.036708 | 7.9596E-06 | 0.123190729 |
| Recessive | 3 | go_ccr2_chemokine_receptor_binding | 3 | 2.0465 | 0.48952 | 0.000014622 | 0.226304694 |
| Recessive | 4 | go_regulation_of_gtpase_activity | 403 | 0.17143 | 0.042371 | 0.000026171 | 0.405048567 |
| Recessive | 5 | reactome_activated_ntrk2_signals_through_frs2_and_frs3 | 7 | 1.2454 | 0.31559 | 0.000039858 | 0.616882266 |
| Recessive | 6 | go_paracrine_signaling | 7 | 1.0674 | 0.28324 | 0.000082394 | 1 |
| Recessive | 7 | reactome_activated_ntrk2_signals_through_ras | 6 | 1.2869 | 0.34411 | 0.000092429 | 1 |
| Recessive | 8 | go_proton_transporting_v_type_atpase_complex_assembly | 3 | 1.562 | 0.41886 | 0.0000964 | 1 |
| Recessive | 9 | go_negative_regulation_of_epidermis_development | 14 | 0.7413 | 0.20624 | 0.00016308 | 1 |
| Recessive | 10 | go_positive_regulation_of_gtpase_activity | 339 | 0.16384 | 0.04581 | 0.00017459 | 1 |
| Recessive | 11 | go_regulation_of_collateral_sprouting | 19 | 0.76139 | 0.21529 | 0.00020331 | 1 |
| Recessive | 12 | holleman_asparaginase_resistance_all_dn | 21 | 0.60371 | 0.174 | 0.00026137 | 1 |
| Recessive | 13 | go_negative_regulation_of_epidermal_cell_differentiation | 11 | 0.77665 | 0.22385 | 0.00026142 | 1 |
| Recessive | 14 | go_positive_regulation_of_nad_p_h_oxidase_activity | 6 | 1.0083 | 0.29626 | 0.00033337 | 1 |
| Recessive | 15 | go_second_messenger_mediated_signaling | 383 | 0.15069 | 0.044776 | 0.00038295 | 1 |
| Recessive | 16 | go_gtpase_regulator_activity | 253 | 0.1766 | 0.052581 | 0.00039269 | 1 |
| Recessive | 17 | reactome_downstream_signaling_of_activated_fgfr1 | 24 | 0.56741 | 0.16896 | 0.00039304 | 1 |
| Recessive | 18 | go_positive_regulation_of_reactive_oxygen_species_metabolic_process | 39 | 0.42711 | 0.12801 | 0.00042524 | 1 |
| Recessive | 19 | reactome_downstream_signaling_of_activated_fgfr3 | 18 | 0.68571 | 0.20553 | 0.00042541 | 1 |
| Recessive | 20 | go_regulation_of_calcineurin_mediated_signaling | 30 | 0.53138 | 0.16015 | 0.00045434 | 1 |

Model, the genetic model in which candidate gene sets were selected by analysis; nGenes, number of genes in the data that are in the gene set; Beta, regression coefficient of the gene set; SE, standard error of the regression coefficient; $p^a$, adjusted *p* value for multiple testing. *Significant association after conservative Bonferroni correction.

**Table S8.** Top 20 candidate gene sets selected from gene-set analysis for the 0-6 h effect site MEC.

| Model | Rank | Gene set name | nGenes | Beta | SE | *p* | *p* [a] |
|---|---|---|---|---|---|---|---|
| Additive | 1 | go_ccr2_chemokine_receptor_binding | 3 | 2.3443 | 0.49002 | 8.6655E-07 | 0.013416794* |
| Additive | 2 | go_paracrine_signaling | 7 | 1.3091 | 0.28351 | 1.9572E-06 | 0.030303328* |
| Additive | 3 | go_sensory_perception_of_taste | 53 | 0.57098 | 0.12678 | 3.3609E-06 | 0.052036815 |
| Additive | 4 | reactome_free_fatty_acid_receptors | 3 | 1.7596 | 0.40156 | 5.9188E-06 | 0.09164078 |
| Additive | 5 | go_taste_receptor_activity | 20 | 1.0332 | 0.23783 | 7.0253E-06 | 0.10877272 |
| Additive | 6 | go_regulation_of_isomerase_activity | 7 | 0.96644 | 0.23857 | 0.000025627 | 0.396782841 |
| Additive | 7 | go_atp_dependent_3_5_dna_helicase_activity | 10 | 0.83307 | 0.20898 | 0.000033691 | 0.521637753 |
| Additive | 8 | go_detection_of_chemical_stimulus_involved_in_sensory_perception_of_taste | 34 | 0.63114 | 0.161 | 0.00004444 | 0.68806452 |
| Additive | 9 | go_calcineurin_mediated_signaling | 40 | 0.52495 | 0.13774 | 0.000069409 | 1 |
| Additive | 10 | go_small_gtpase_mediated_signal_transduction | 522 | 0.13619 | 0.035987 | 0.000077335 | 1 |
| Additive | 11 | go_rna_secondary_structure_unwinding | 8 | 1.0748 | 0.2875 | 0.000092889 | 1 |
| Additive | 12 | go_positive_regulation_of_binding | 168 | 0.22674 | 0.060821 | 0.000096826 | 1 |
| Additive | 13 | go_nuclear_envelope_lumen | 9 | 1.0235 | 0.28052 | 0.00013233 | 1 |
| Additive | 14 | go_3_5_rna_helicase_activity | 19 | 0.58181 | 0.16123 | 0.00015444 | 1 |
| Additive | 15 | sotiriou_breast_cancer_grade_1_vs_3_dn | 48 | 0.37133 | 0.10425 | 0.00018455 | 1 |
| Additive | 16 | go_sensory_perception_of_chemical_stimulus | 323 | 0.20535 | 0.057653 | 0.00018464 | 1 |
| Additive | 17 | go_urinary_tract_smooth_muscle_contraction | 6 | 1.2814 | 0.36675 | 0.0002387 | 1 |
| Additive | 18 | go_motor_activity | 128 | 0.2539 | 0.073102 | 0.00025784 | 1 |
| Additive | 19 | go_regulation_of_binding | 335 | 0.15189 | 0.043882 | 0.00026955 | 1 |
| Additive | 20 | go_intrinsic_apoptotic_signaling_pathway_in_response_to_dna_damage_by_p53_clas | 34 | 0.49439 | 0.14362 | 0.00028895 | 1 |
| Dominant | 1 | dazard_uv_response_cluster_g5 | 9 | 0.87321 | 0.21343 | 0.000021542 | 0.333491702 |
| Dominant | 2 | go_ciliary_base | 34 | 0.52323 | 0.13273 | 0.000040545 | 0.627677145 |
| Dominant | 3 | hofmann_myelodysplastic_syndrom_high_risk_up | 8 | 0.86364 | 0.22403 | 0.000058086 | 0.899229366 |
| Dominant | 4 | biocarta_akapcentrosome_pathway | 14 | 0.7873 | 0.21402 | 0.00011763 | 1 |
| Dominant | 5 | biocarta_akap95_pathway | 11 | 0.8784 | 0.24077 | 0.00013239 | 1 |
| Dominant | 6 | biocarta_akap13_pathway | 11 | 0.86161 | 0.24075 | 0.00017301 | 1 |
| Dominant | 7 | biocarta_nos1_pathway | 21 | 0.65274 | 0.18246 | 0.00017402 | 1 |
| Dominant | 8 | biocarta_csk_pathway | 20 | 0.65229 | 0.18499 | 0.00021141 | 1 |
| Dominant | 9 | wallace_jak2_targets_up | 21 | 0.57998 | 0.16606 | 0.00023979 | 1 |
| Dominant | 10 | biocarta_agpcr_pathway | 11 | 0.83868 | 0.24132 | 0.00025571 | 1 |
| Dominant | 11 | go_cluster_of_actin_based_cell_projections | 140 | 0.23865 | 0.070595 | 0.00036249 | 1 |
| Dominant | 12 | go_nucleotide_activated_protein_kinase_complex | 13 | 0.70679 | 0.21315 | 0.00045765 | 1 |
| Dominant | 13 | go_tooth_mineralization | 21 | 0.61937 | 0.1878 | 0.00048778 | 1 |
| Dominant | 14 | go_macrophage_colony_stimulating_factor_production | 5 | 1.2955 | 0.39293 | 0.00048943 | 1 |
| Dominant | 15 | biocarta_gpcr_pathway | 27 | 0.49538 | 0.15171 | 0.00054794 | 1 |
| Dominant | 16 | biocarta_tcytotoxic_pathway | 12 | 0.78553 | 0.24939 | 0.00081832 | 1 |
| Dominant | 17 | go_substantia_nigra_development | 38 | 0.41065 | 0.13067 | 0.0008385 | 1 |
| Dominant | 18 | abdulrahman_kidney_cancer_vhl_dn | 13 | 0.78408 | 0.25131 | 0.00090584 | 1 |
| Dominant | 19 | go_enamel_mineralization | 16 | 0.70933 | 0.22788 | 0.0009286 | 1 |
| Dominant | 20 | go_positive_regulation_of_chromatin_binding | 13 | 0.71356 | 0.23015 | 0.00096771 | 1 |
| Recessive | 1 | go_paracrine_signaling | 7 | 1.371 | 0.28345 | 6.6572E-07 | 0.010303348* |
| Recessive | 2 | go_ccr2_chemokine_receptor_binding | 3 | 2.2819 | 0.48996 | 1.6149E-06 | 0.024993807* |
| Recessive | 3 | go_small_gtpase_mediated_signal_transduction | 495 | 0.155 | 0.036748 | 0.000012389 | 0.191744553 |
| Recessive | 4 | reactome_free_fatty_acid_receptors | 3 | 1.6498 | 0.40153 | 0.000019985 | 0.309307845 |
| Recessive | 5 | kegg_peroxisome | 72 | 0.34912 | 0.087875 | 0.000035667 | 0.552018159 |
| Recessive | 6 | reactome_beta_oxidation_of_pristanoyl_coa | 9 | 0.8723 | 0.2229 | 0.000045707 | 0.707407239 |
| Recessive | 7 | reactome_activated_ntrk2_signals_through_frs2_and_frs3 | 7 | 1.2124 | 0.31593 | 0.000062391 | 0.965625507 |
| Recessive | 8 | go_positive_regulation_of_protein_binding | 84 | 0.32297 | 0.084698 | 0.000068864 | 1 |
| Recessive | 9 | go_nuclear_envelope_lumen | 9 | 1.046 | 0.28047 | 0.000096328 | 1 |
| Recessive | 10 | go_positive_regulation_of_binding | 162 | 0.22661 | 0.061747 | 0.00012172 | 1 |
| Recessive | 11 | lee_liver_cancer_acox1_up | 55 | 0.41497 | 0.11472 | 0.00014936 | 1 |
| Recessive | 12 | go_protein_glycosylation_in_golgi | 4 | 1.2619 | 0.35233 | 0.00017132 | 1 |
| Recessive | 13 | reactome_downstream_signaling_of_activated_fgfr1 | 24 | 0.6042 | 0.16912 | 0.00017733 | 1 |
| Recessive | 14 | go_negative_regulation_of_jnk_cascade | 33 | 0.47435 | 0.13288 | 0.00017922 | 1 |
| Recessive | 15 | reactome_signaling_by_fgfr_in_disease | 53 | 0.40712 | 0.11465 | 0.00019238 | 1 |
| Recessive | 16 | reactome_signaling_by_fgfr1_in_disease | 32 | 0.51221 | 0.14563 | 0.00021872 | 1 |
| Recessive | 17 | reactome_activated_ntrk2_signals_through_ras | 6 | 1.2095 | 0.34448 | 0.00022379 | 1 |
| Recessive | 18 | go_vascular_endothelial_cell_proliferation | 15 | 0.75537 | 0.21628 | 0.00023995 | 1 |
| Recessive | 19 | reactome_downstream_signaling_of_activated_fgfr3 | 18 | 0.7185 | 0.20573 | 0.00024006 | 1 |
| Recessive | 20 | go_fatty_acid_beta_oxidation_using_acyl_coa_oxidase | 14 | 0.60977 | 0.17529 | 0.00025272 | 1 |

Model, the genetic model in which candidate gene sets were selected by analysis; nGenes, number of genes in the data that are in the gene set; Beta, regression coefficient of the gene set; SE, standard error of the regression coefficient; $p^a$, adjusted $p$ value for multiple testing. *Significant association after conservative Bonferroni correction.

**Table S9.** Top 20 candidate gene sets selected from gene-set analysis for the 0-12 h effect site MEC.

| Model | Rank | Gene set name | nGenes | Beta | SE | *p* | *p* [a] |
|---|---|---|---|---|---|---|---|
| Additive | 1 | go_ccr2_chemokine_receptor_binding | 3 | 2.1567 | 0.48839 | 5.0629E-06 | 0.078388881 |
| Additive | 2 | pid_shp2_pathway | 53 | 0.51371 | 0.11648 | 0.000005191 | 0.080372253 |
| Additive | 3 | go_small_gtpase_mediated_signal_transduction | 522 | 0.147 | 0.03586 | 0.000020828 | 0.322479924 |
| Additive | 4 | go_positive_regulation_of_binding | 168 | 0.239 | 0.060609 | 0.00004036 | 0.62489388 |
| Additive | 5 | go_positive_regulation_of_superoxide_anion_generation | 16 | 0.72254 | 0.18328 | 0.000040533 | 0.627572439 |
| Additive | 6 | sotiriou_breast_cancer_grade_1_vs_3_dn | 48 | 0.39274 | 0.10388 | 0.000078503 | 1 |
| Additive | 7 | kegg_amyotrophic_lateral_sclerosis_als | 48 | 0.42908 | 0.1169 | 0.00012151 | 1 |
| Additive | 8 | go_calcineurin_mediated_signaling | 40 | 0.49515 | 0.13727 | 0.0001553 | 1 |
| Additive | 9 | reactome_free_fatty_acid_receptors | 3 | 1.3747 | 0.40027 | 0.00029773 | 1 |
| Additive | 10 | go_urinary_tract_smooth_muscle_contraction | 6 | 1.2532 | 0.36549 | 0.00030392 | 1 |
| Additive | 11 | go_regulation_of_isomerase_activity | 7 | 0.81399 | 0.23778 | 0.00031017 | 1 |
| Additive | 12 | go_positive_regulation_of_protein_binding | 87 | 0.28252 | 0.082807 | 0.00032344 | 1 |
| Additive | 13 | reactome_activated_ntrk2_signals_through_frs2_and_frs3 | 7 | 1.0744 | 0.31492 | 0.00032372 | 1 |
| Additive | 14 | go_taste_receptor_activity | 20 | 0.80835 | 0.23707 | 0.00032586 | 1 |
| Additive | 15 | go_positive_regulation_of_nad_p_h_oxidase_activity | 6 | 1.0078 | 0.29559 | 0.00032613 | 1 |
| Additive | 16 | holleman_asparaginase_resistance_all_dn | 22 | 0.58464 | 0.17149 | 0.00032669 | 1 |
| Additive | 17 | go_positive_regulation_of_posttranscriptional_gene_silencing | 22 | 0.57318 | 0.16888 | 0.00034525 | 1 |
| Additive | 18 | go_regulation_of_rna_polymerase_ii_transcriptional_preinitiation_complex_assembly | 14 | 0.78618 | 0.23217 | 0.00035524 | 1 |
| Additive | 19 | go_paracrine_signaling | 7 | 0.95584 | 0.28262 | 0.00036066 | 1 |
| Additive | 20 | go_nuclear_envelope_lumen | 9 | 0.9446 | 0.27957 | 0.0003649 | 1 |
| Dominant | 1 | hofmann_myelodysplastic_syndrom_high_risk_up | 8 | 0.88164 | 0.22246 | 0.000037152 | 0.575150112 |
| Dominant | 2 | reactome_clec7a_dectin_1_induces_nfat_activation | 11 | 0.99528 | 0.27286 | 0.00013278 | 1 |
| Dominant | 3 | dazard_uv_response_cluster_g5 | 9 | 0.75989 | 0.21196 | 0.00016901 | 1 |
| Dominant | 4 | go_tooth_mineralization | 21 | 0.66801 | 0.18648 | 0.0001708 | 1 |
| Dominant | 5 | go_gator2_complex | 10 | 0.87496 | 0.24889 | 0.00022013 | 1 |
| Dominant | 6 | biocarta_akap13_pathway | 11 | 0.83466 | 0.23907 | 0.00024101 | 1 |
| Dominant | 7 | go_cluster_of_actin_based_cell_projections | 140 | 0.24365 | 0.070102 | 0.00025542 | 1 |
| Dominant | 8 | biocarta_gpcr_pathway | 27 | 0.51709 | 0.15065 | 0.0002999 | 1 |
| Dominant | 9 | go_nucleotide_activated_protein_kinase_complex | 13 | 0.72636 | 0.21166 | 0.00030057 | 1 |
| Dominant | 10 | go_myristoyl_coa_hydrolase_activity | 5 | 1.2325 | 0.36237 | 0.00033639 | 1 |
| Dominant | 11 | go_enamel_mineralization | 16 | 0.76313 | 0.22628 | 0.00037338 | 1 |
| Dominant | 12 | biocarta_nos1_pathway | 21 | 0.60135 | 0.1812 | 0.00045317 | 1 |
| Dominant | 13 | biocarta_akap95_pathway | 11 | 0.76923 | 0.23911 | 0.00064886 | 1 |
| Dominant | 14 | biocarta_csk_pathway | 20 | 0.57789 | 0.18371 | 0.00083013 | 1 |
| Dominant | 15 | biocarta_akapcentrosome_pathway | 14 | 0.66609 | 0.21255 | 0.00086441 | 1 |
| Dominant | 16 | go_seh1_associated_complex | 13 | 0.68982 | 0.22079 | 0.00089243 | 1 |
| Dominant | 17 | go_atp_binding_cassette_abc_transporter_complex | 7 | 1.0128 | 0.33132 | 0.0011207 | 1 |
| Dominant | 18 | go_ciliary_base | 34 | 0.4023 | 0.13183 | 0.0011396 | 1 |
| Dominant | 19 | go_regulation_of_cofactor_metabolic_process | 31 | 0.4247 | 0.13955 | 0.0011717 | 1 |
| Dominant | 20 | go_corpus_callosum_morphogenesis | 6 | 1.0017 | 0.33009 | 0.0012064 | 1 |
| Recessive | 1 | pid_shp2_pathway | 52 | 0.5572 | 0.12035 | 1.8442E-06 | 0.028542683* |
| Recessive | 2 | go_ccr2_chemokine_receptor_binding | 3 | 2.1236 | 0.48984 | 7.3264E-06 | 0.113390693 |
| Recessive | 3 | go_small_gtpase_mediated_signal_transduction | 495 | 0.15804 | 0.036734 | 8.5031E-06 | 0.131602479 |
| Recessive | 4 | go_proton_transporting_v_type_atpase_complex_assembly | 3 | 1.7214 | 0.41911 | 0.000020119 | 0.311381763 |
| Recessive | 5 | reactome_activated_ntrk2_signals_through_frs2_and_frs3 | 7 | 1.2966 | 0.3158 | 0.000020249 | 0.313393773 |
| Recessive | 6 | go_regulation_of_gtpase_activity | 403 | 0.16507 | 0.042402 | 0.000049727 | 0.769624779 |
| Recessive | 7 | reactome_activated_ntrk2_signals_through_ras | 6 | 1.3283 | 0.34433 | 0.000057478 | 0.889587006 |
| Recessive | 8 | go_positive_regulation_of_nad_p_h_oxidase_activity | 6 | 1.1279 | 0.29643 | 0.000071241 | 1 |
| Recessive | 9 | go_urinary_tract_smooth_muscle_contraction | 6 | 1.3825 | 0.36654 | 0.000081376 | 1 |
| Recessive | 10 | go_paracrine_signaling | 7 | 1.0367 | 0.28344 | 0.00012781 | 1 |
| Recessive | 11 | go_positive_regulation_of_protein_binding | 84 | 0.30269 | 0.084673 | 0.00017575 | 1 |
| Recessive | 12 | go_positive_regulation_of_binding | 162 | 0.2203 | 0.061727 | 0.0001797 | 1 |
| Recessive | 13 | go_positive_regulation_of_superoxide_anion_generation | 16 | 0.63913 | 0.18384 | 0.00025474 | 1 |
| Recessive | 14 | go_positive_regulation_of_reactive_oxygen_species_metabolic_process | 39 | 0.44027 | 0.1281 | 0.0002949 | 1 |
| Recessive | 15 | go_positive_regulation_of_gtpase_activity | 339 | 0.15718 | 0.045843 | 0.00030396 | 1 |
| Recessive | 16 | go_second_messenger_mediated_signaling | 383 | 0.15236 | 0.044807 | 0.00033734 | 1 |
| Recessive | 17 | go_g_protein_coupled_serotonin_receptor_binding | 7 | 1.0807 | 0.31964 | 0.00036206 | 1 |
| Recessive | 18 | go_nuclear_envelope_lumen | 9 | 0.94573 | 0.2804 | 0.0003729 | 1 |
| Recessive | 19 | reactome_downstream_signaling_of_activated_fgfr1 | 24 | 0.57002 | 0.16907 | 0.00037478 | 1 |
| Recessive | 20 | go_regulation_of_collateral_sprouting | 19 | 0.72229 | 0.21545 | 0.00040148 | 1 |

Model, the genetic model in which candidate gene sets were selected by analysis; nGenes, number of genes in the data that are in the gene set; Beta, regression coefficient of the gene set; SE, standard error of the regression coefficient; $p^a$, adjusted $p$ value for multiple testing. *Significant association after conservative Bonferroni correction.

**Table S10.** Genes included in the best candidate gene sets selected from gene-set analyses.

| Gene set name | Gene (alphabetical order) |
|---|---|
| go_paracrine_signaling | *CD34, CGAS, FGF2, PDGFB**, *PGR, SERPINB3, TNFSF11* |
| reactome_free_fatty_acid_receptors | *FFAR1, FFAR2, FFAR3, FFAR4**, *GPR31* |
| go_taste_receptor_activity | *FFAR4**, *PKD1L3, PKD2L1, TAS1R1, TAS1R2, TAS1R3, TAS2R1, TAS2R10, TAS2R13, TAS2R14, TAS2R16, TAS2R19, TAS2R20, TAS2R3, TAS2R30, TAS2R31, TAS2R38, TAS2R39, TAS2R4, TAS2R40, TAS2R41, TAS2R42, TAS2R43, TAS2R45, TAS2R46, TAS2R5, TAS2R50, TAS2R60, TAS2R7, TAS2R8, TAS2R9* |
| go_negative_regulation_of_epidermal_cell_differentiation | *ACVRL1, CAV1, CCND1, CTNNB1, DLL1**, *EZH2***, *FGF10, FOXE3, FOXJ2, FRZB, FST, GDF3**, *GRHL2***, *HES1**, *HES5**, *HOXA7***, *ID1, IFNG**, *IL13, IL1A, JAG1, KRAS**, *MIR1-1, MIR10A, MIR18B, MIR29B1, MIR495, MIR518B, MMP9, MSX2***, *NKX6-3, NODAL, NOTCH1**, *NOTCH4, OSR1, REG3A***, *REG3G***, *S1PR3, SIX2, SMO, SOX9, SPRED1, SPRED2, SPRED3, SPRY1, SPRY2, SRSF6***, *STAT1**, *TBX3, TP63***, *VEGFA**, *XDH, YAP1, ZEB1* |
| go_negative_regulation_of_epidermis_development | *DLL1**, *EZH2***, *GDF3**, *GRHL2***, *HES1**, *HES5**, *HOXA7***, *MSX2***, *NOTCH1**, *REG3A***, *REG3G***, *SRSF6***, *TP63*** |
| go_negative_regulation_of_keratinocyte_differentiation | *EZH2***, *GRHL2***, *HOXA7***, *MSX2***, *REG3A***, *REG3G***, *SRSF6***, *TP63*** |
| sotiriou_breast_cancer_grade_1_vs_3_dn | *AASS, AKAP11, ARHGEF12, ARHGEF40, BBOF1, BBS1, CASP9, CFAP69, CIRBP, CRTC3, CRY2, CTDSP1, CX3CR1, CYBRD1, DEAF1, DIXDC1, DYNC2H1, DZANK1, ECHDC2, FOS, FRY, IFT46, IFT88, JHY, KIF13B, LAMB2, LTBP3, MARCHF8, MPHOSPH8, NBR1, NF1, NME5, NYNRIN, PIGV, RGPD5, RNASE4, RUNX1, SESN1, SIRT3, SLC24A1, SMARCA2, SNX1, STARD13, STAT5B, SYNC, TBC1D17, TP53BP1, TP73-AS1, TPT1, WDR19, ZFP2, ZNF395, ZNF862* |
| go_ccr2_chemokine_receptor_binding | *CCL2, CCL7, CCR2, DEFB106A, DEFB106B, MSMP* |
| pid_shp2_pathway | *AFDN, ANGPT1, ARHGAP35, BDNF, EGF, EGFR, FRS2, FRS3, GAB1, GAB2, GNAI1, GNAI3, GRB2, HRAS, IFNG**, *IFNGR1, IGF1R, IL2, IL2RA, IL2RB, IL2RG, IL6, IL6R, IL6ST, IRS1, JAK1, JAK2, JAK3, KDR, KRAS**, *LCK, LMO4, MAP2K1, MAP2K2, NGF, NOS3, NRAS, NTF3, NTF4, NTRK1, NTRK2, NTRK3, PAG1, PDGFB**, *PDGFRB, PIK3CA, PIK3R1, PRKACA, PTPN11, RAF1, RHOA, SDC2, SHC1, SOS1, STAT1**, *TEK, VEGFA** |

*, genes commonly included in two kinds of gene sets; **, genes commonly included in three kinds of gene sets.

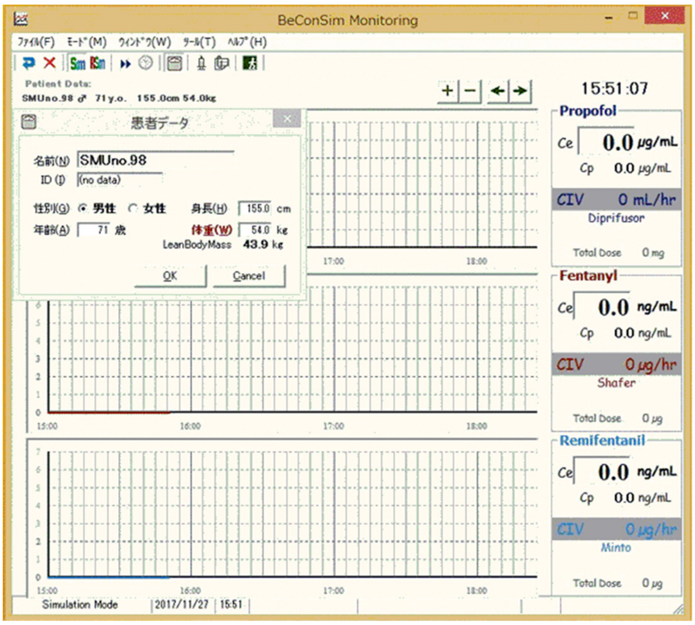


**Figure S4.** Snapshot of BeConSim Monitoring software for the calculation of estimated plasma and effect site concentrations of fentanyl.

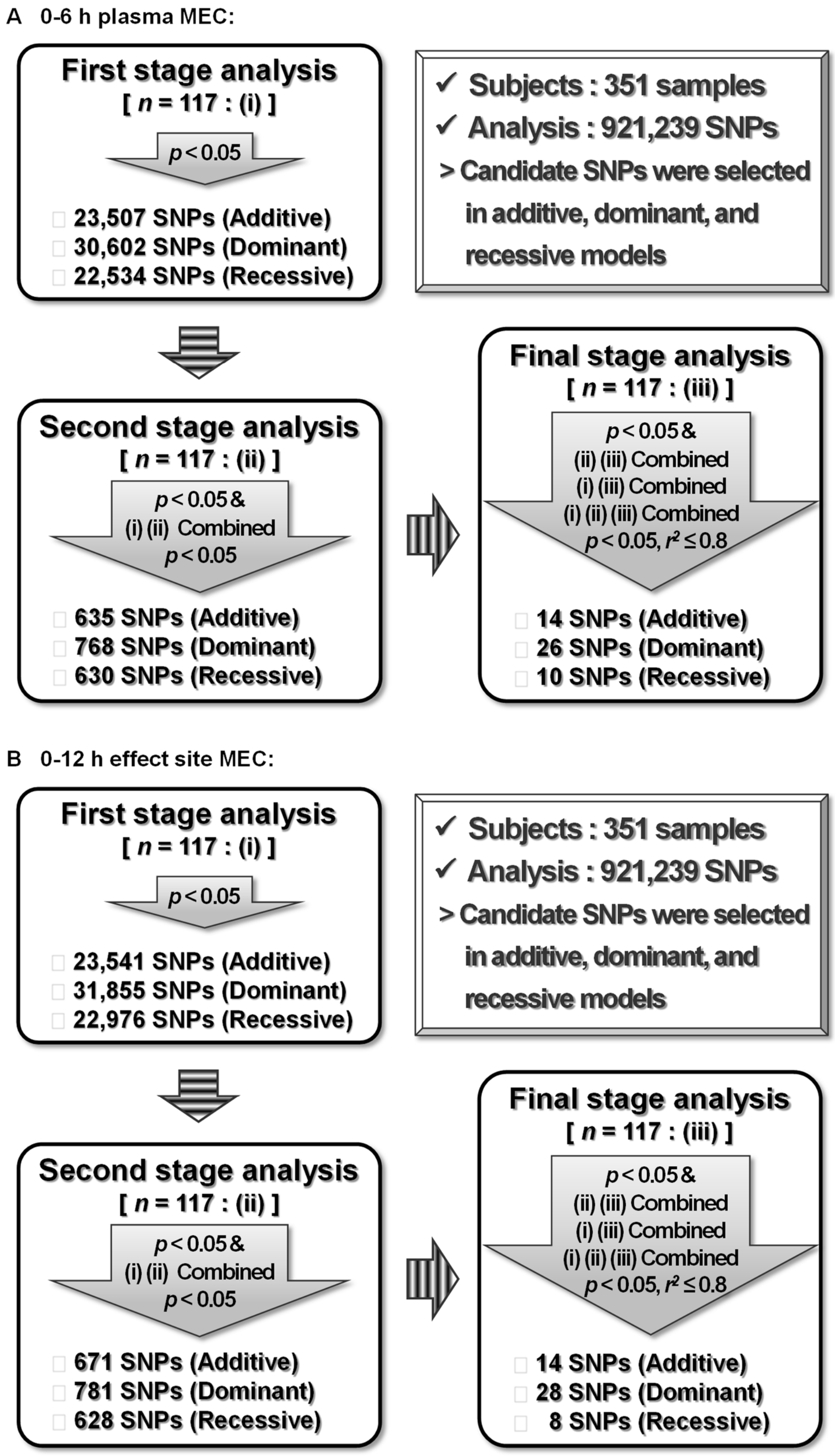


**Figure S5.** Schematic illustration of the multistage GWAS. Candidate SNPs associated with human opioid sensitivity were selected in a three-stage GWAS. (**A**) Illustration of the GWAS for the 0-6 h plasma MEC. (**B**) Illustration of the GWAS for the 0-12 h effect site MEC.
